# Magnetic Fluorescent Quantum Dots Nanocomposites in Food Contaminants Analysis: Current Challenges and Opportunities

**DOI:** 10.3390/ijms23084088

**Published:** 2022-04-07

**Authors:** Jincheng Xiong, Huixia Zhang, Linqian Qin, Shuai Zhang, Jiyue Cao, Haiyang Jiang

**Affiliations:** 1 Department of Veterinary Pharmacology and Toxicology, College of Veterinary Medicine, China Agricultural University, Beijing Key Laboratory of Detection Technology for Animal-Derived Food Safety, Beijing Laboratory for Food Quality and Safety, Beijing 100193, China; xiongjincheng94@163.com (J.X.); zhanghuixia2008@163.com (H.Z.); s20213050796@cau.edu.cn (L.Q.); zxxxsc@163.com (S.Z.); 2 Department of Veterinary Pharmacology, College of Veterinary Medicine, Huazhong Agricultural University, Wuhan 430070, China; caojiyue@mail.hzau.edu.cn

**Keywords:** magnetic nanoparticles, quantum dots, magnetic quantum dots, nanocomposites, food contaminants analysis

## Abstract

The presence of food contaminants can cause foodborne illnesses, posing a severe threat to human health. Therefore, a rapid, sensitive, and convenient method for monitoring food contaminants is eagerly needed. The complex matrix interferences of food samples and poor performance of existing sensing probes bring significant challenges to improving detection performances. Nanocomposites with multifunctional features provide a solution to these problems. The combination of the superior characteristics of magnetic nanoparticles (MNPs) and quantum dots (QDs) to fabricate magnetic fluorescent quantum dots (MNPs@QDs) nanocomposites are regarded as an ideal multifunctional probe for food contaminants analysis. The high-efficiency pretreatment and rapid fluorescence detection are concurrently integrated into one sensing platform using MNPs@QDs nanocomposites. In this review, the contemporary synthetic strategies to fabricate MNPs@QDs, including hetero-crystalline growth, template embedding, layer-by-layer assembly, microemulsion technique, and one-pot method, are described in detail, and their advantages and limitations are discussed. The recent advances of MNPs@QDs nanocomposites in detecting metal ions, foodborne pathogens, toxins, pesticides, antibiotics, and illegal additives are comprehensively introduced from the perspectives of modes and detection performances. The review ends with current challenges and opportunities in practical applications and prospects in food contaminants analysis, aiming to promote the enthusiasm for multifunctional sensing platform research.

## 1. Introduction

Food is the essential material basis for the survival of human beings, unsafe food seriously damages human health and affects social and economic development. The existence of food contaminants is one of the major factors endangering food safety and human health, and the extension of modern food production and processing industry chain also increases the potential risk of food contamination. Even if trace contaminants enter the food chain, they will also cause severe health risks by the bioaccumulation effect [1,2,3]. Therefore, the analysis methods characterized by simple, low-cost, rapid, and sensitive for food contaminants detection are urgently demanded.

One of the primary goals of modern analytical techniques is to develop sensitive, accurate, reliable, and high-throughput food safety detection and quality assurance methods to identify, monitor, and quantify newly discovered food components and contaminants [3,4]. At present, multiple mature instrument analytical techniques have been employed to detect various food contaminants [5,6,7]. However, the requirements of sophisticated devices, complex pretreatment processes, and professional operators make them unsuitable for rapid on-site screening of many samples. The development of nanotechnology has provided a robust basis for the design of nanomaterials with unique physicochemical properties, and it offers an opportunity to establish analysis platforms for the separation, detection, and tracing of food contaminants [8].

The development of nanomaterial-based sensors as alternative methods or complementary analytical tools for food contaminations analysis has recently accelerated since they have the merit of being convenient, efficient, and low-cost in on-site screening [9,10,11]. Although versatile sensors are easily accessible and enable wide applications, there are many types of food contaminants with distinct properties, and the concentration level is usually as low as ppm or even ppb, meaning that the matrix effects of food samples are complicated and challenging. Severe interference may produce during the analysis process, which will bring significant challenges to the current sensor technologies [12,13]. Therefore, the two key points to achieve sensitive and accurate food contaminant analysis are (i) elimination of matrices interference and (ii) enrichment of target analytes. Among the numerous attractive nanomaterials, magnetic nanoparticles (MNPs) have attracted widespread attention due to their unique controllability [14]. MNPs have strong magnetic responsiveness, and can be quickly retrieved and separated from complex sample matrices under an external magnetic field [15]. The fantastic properties can be applied as a powerful tool to easily control the capture and release of low concentrations of target analytes in complicated food samples [16]. When the external magnetic field is removed, the magnetism of MNPs disappears rapidly and can be redispersed [17]. Furthermore, most MNPs can be recycled multiple times to avoid pollution, indicating that MNPs are environmentally friendly [18].

The sensors constitute recognition and transducer elements. The recognition elements usually employed antibody [19], aptamer [20], and molecularly imprinted polymers (MIPs) [21], which provide high specificity and selectivity for target analytes. Transducer elements are usually separate chemical or physical sensing elements that convert biological reactions into easily detectable signals. Fluorescent nanomaterials are selected as the transducer element due to their excellent optical properties, which can significantly improve the sensitivity of fluorescence detection [22,23,24,25]. Among the fluorescent nanomaterials used for sensing, quantum dots (QDs) show the advantages of stable physicochemical properties, good biocompatibility, dispersity, and simple surface functionalization [26]. The QDs with a quasi-spherical microstructure have excellent luminescent properties, such as high quantum yield (QY), narrow symmetric and tunable fluorescence spectra, and photobleaching resistance, demonstrating that the QDs are currently attracting enormous attention as promising candidates for transducer elements and improving the sensitivity of sensing analysis [27,28,29,30].

With the development and maturity of QDs, the combination of QDs with MNPs to form multifunctional nanocomposites has attracted increasing interest [31,32,33,34,35,36]. Magnetic fluorescent quantum dots (MNPs@QDs) nanocomposites have both magnetic and fluorescent properties, which can not only quickly enrich and separate the target analytes from the complicated food matrices under the external magnetic field. They also realize the quantitative analysis of the targets, greatly simplifying the pretreatment process. This avoids the loss of the analytes to be tested, appreciably shortening the detection time and improving the detection efficiency. As novel multifunctional nanocomposites, MNPs@QDs have great potential application values and important research significance in food contaminants analysis.

**Figure 1:**
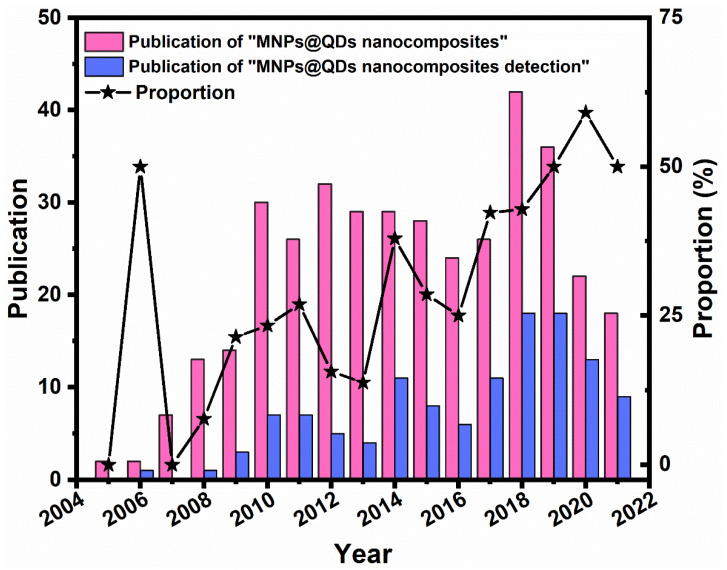
Number of published papers regarding magnetic fluorescent quantum dots nanocomposites research by the end of 2021. The black line represents the proportion of the number of publications on “magnetic fluorescent quantum dots nanocomposites detection” to “magnetic fluorescent quantum dots nanocomposites.” The data was derived from the Web of Science by searching the terms “magnetic fluorescent quantum dots nanocomposites” or “magnetic fluorescent quantum dots nanocomposites detection” (https://app.webofknowledge.com accessed on 31 December 2021).

**Figure 2:**
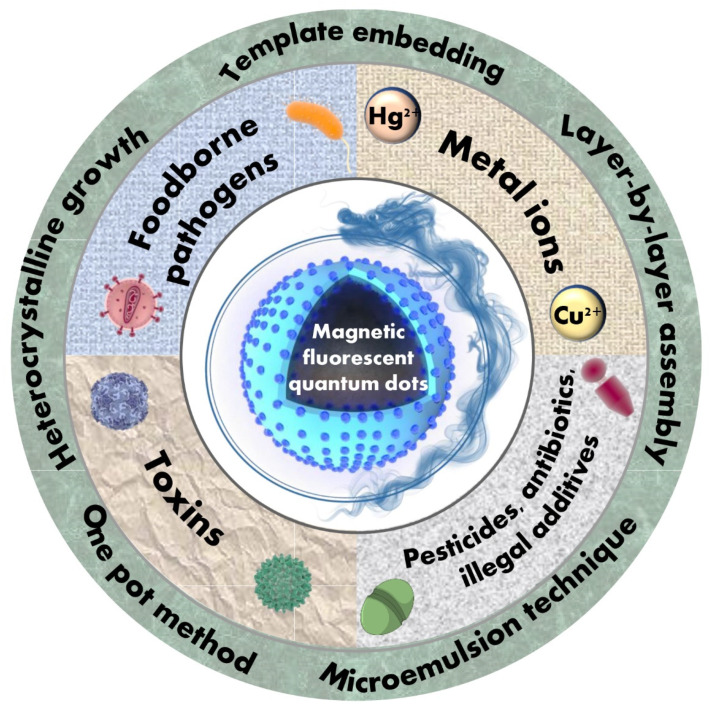
Overview of magnetic fluorescent quantum dots-based sensors for food contaminants analysis.

Over the past 20 years, hundreds of articles have been reported in the literature on the Web of Science with the term “magnetic fluorescent quantum dots nanocomposites (Figure 1).” The works have reported various preparation and characterization methods, and their research advances in various aspects such as bioimaging, drug delivery, environmental monitoring, and biosensing platforms [34,37,38,39], indicating their scientific significance and application prospects. However, to the best of our knowledge, there are still no review articles focusing on the applications research progress of MNPs@QDs nanocomposites in food contaminants analysis. To fill in the information in this area, this review will provide a comprehensive perspective inspecting the current opportunities and challenges of MNPs@QDs in food contaminants analysis. Herein, five commonly used methods for preparing MNPs@QDs are introduced in detail, including hetero-crystalline growth, template embedding, layer-by-layer (LBL) assembly, microemulsion technique, and the one-pot method. The design strategy of core-shell structure MNPs@QDs nanocomposites and the critical points of its application are discussed. The recent advances of MNPs@QDs nanocomposites application in detecting metal ions, foodborne pathogens, toxins, pesticides, antibiotics, and illegal additives are introduced in terms of their modes and detection performances. Finally, we propose the current challenges and opportunities in food contaminants analysis and look forward to future application prospects in food safety (Figure 2).

## 2. Preparation of MNPs@QDs

Currently, the preparation methods of MNPs@QDs have been reported in numerous pieces of the literature [34,38]. The key point to consider in the reasonable integration of MNPs and QDs into multifunctional MNPs@QDs nanocomposites is whether the two components are firmly combined and interact without causing a loss of magnetic and fluorescent performance. Furthermore, its properties such as dispersibility, stability, biocompatibility, and desirable surface functionalization are also related to its practical applications. Although the compositions and morphologies of these nanocomposites perform differently, most strategies can be divided into five categories: hetero-crystalline growth, template embedding, LBL assembly, microemulsion technique, and the one-pot method.

### 2.1. Hetero-Crystalline Growth

**Figure 3:**
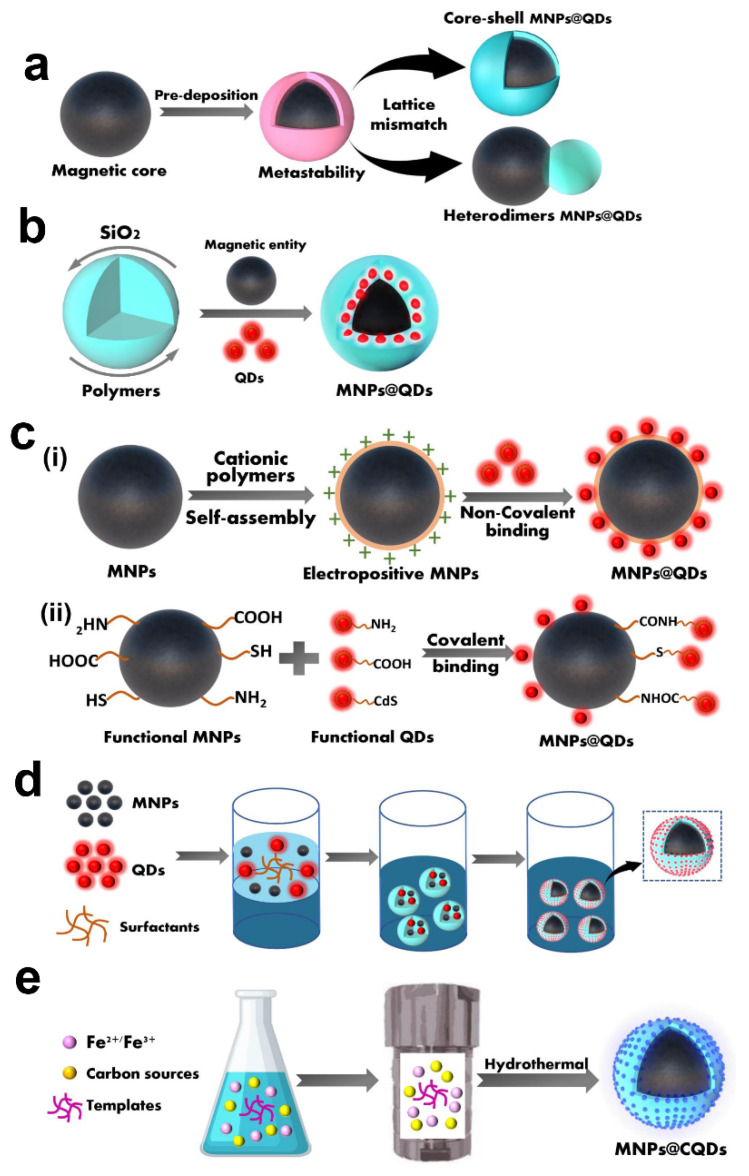
Schematic diagram of five preparation methods of MNPs@QDs nanocomposites. (**a**) Hetero-crystalline growth; (**b**) Template embedding; (**c**) Layer-by-layer assembly constitute of (**i**) non-covalent binding and (**ii**) covalent binding; (**d**) Microemulsion technique; (**e**) One-pot method.

Hetero-crystalline growth usually combines MNPs and QDs in either core-shell or two asymmetric nanoparticles (heterodimers). The deposition of semiconductor materials on the prefabricated magnetic nanocrystals by decomposing the precursors at high temperatures generate the formation of MNPs@QDs nanocomposites with distinct functional domains. The typical core-shell structures were reported such as Co@CdSe [40], Fe_3_O_4_/CdSe/ZnS [41,42], and Fe_3_O_4_@PANI/CQDs [43]; and heterodimer structures such as FePt-Pb (S, Se) [44], FePt@CdS [45], Fe_2_O_3_-CdSe [46,47], Fe_3_O_4_-CdS (Se) [48,49]. Gu et al. reported a simple synthesis route for producing heterodimer nanoparticles by employing lattice mismatching and high-temperature decomposition [45]. In the presence of oleylamine and oleic acid, Fe@Pt was firstly formed at high-temperature decomposition. Subsequently, S and Cd were successively deposited on the surface of Fe@Pt to form metastable core-shell nanostructures at 100 °C. With the increase of solution temperature to 280 °C, the amorphous CdS on the sphere’s surface was transformed into a crystal and formed heterodimers FePt@CdS nanoparticles with an appropriate size of 7 nm and a QY of 3.2% (Figure 3a) The core-shell or heterodimer MNPs@QDs all strongly depend on the lattice mismatch between the MNPs and QDs components, and synthesis conditions such as the reaction temperature, surface capping agents and the addition order of the precursors [44,45].

Although the hetero-crystalline growth method was first reported for preparing MNP@QDs, the main problems of the method are the inability to optimize the properties of the nanocomposites, undesirable magnetic responsiveness, low QY, and lack of functional groups, which make it challenging to satisfy the diversified requirements of food contaminants analysis. The initial nanocrystals can be prepared under optimal conditions; however, in the preparation of subsequent components, the initial crystals may be subject to a series of temperature variations that affect their structure. Moreover, the undesirable interfacial interaction between MNPs and QDs may cause the loss of the dual performances of MNPs@QDs. The inner filter effect (IFE) (non-radioactive quenching) induced by MNPs and the poor crystal quality of QDs lead to the QY of MNPs@QDs usually less than 10%. For example, after passivating the surface layer of Fe_3_O_4_@CdSe by ZnS, the QY was increased from 2–3% to 10–15% [50]. This value was still much lower than that of standard CdSe/ZnS QDs alone [51]. The magnetism of MNPs is relatively easy to maintain, but interfacial doping or instability between two lattices may result in disorder in MNPs structure [52]; it is still a troublesome problem in the currently reported research by hetero-crystalline growth method.

### 2.2. Template Embedding

Template embedding usually encapsulates the discrete preformed MNPs and QDs into the liposomes [53], micelles [54,55], silica [56,57,58,59,60,61,62], and polymer materials [63,64,65,66,67] simultaneously to obtain MNPs@QDs. The spatial separation between MNPs and QDs helps avoid the mutual interference between the two components, reducing the possibility of reduced magnetic responsiveness or fluorescence quenching.

Silica (SiO_2_) has the advantages of good biological inertia and biocompatibility, facile synthesis, and operable surface functionalization, which offer a favorable carrier for loading MNPs and QDs to form MNPs@QDs nanocomposites. Dong et al. encapsulated both Fe_2_O_3_ and CdSe QDs within a silica shell to form SiO_2_/MNPs-QDs. The QY decreased appropriately 4- and 10-fold lower than bare CdSe QDs (QY = 11.4%) at the coating reacting at 8 and 48 h, respectively [59]. The quenching effect of MNPs and multiple chemical reactions still inevitably affects the dual performance of nanocomposites, and seeking better spatial separation is significant for its further application. The silica layer wraps on the surface of the MNPs as a classical strategy to construct an efficient barrier to prevent fluorescence quenching by adjusting the thickness of the silica shell. Easy manipulation of surface functionalization provides plenty of binding sites for QDs and recognition elements conjugation. Furthermore, the silica coating helps to reduce the toxicity of bare MNPs, and improve their stability and dispersity, which is conducive to subsequent biological applications [61,68,69,70]. Meanwhile, the hollow and mesoporous silica templates can effectively reduce the density of the silica, enhance the transmissivity of irradiation light, and avoid the side effects of absorption and scattering [56,57,62,71]. The general strategy of template embedment is illustrated in Figure 3b.

In addition to SiO_2_, polymer materials are also employed as carriers for encapsulating MNPs and QDs, which is commonly achieved by hydrophobic [64,65,72], electrostatic [73], and covalent [74,75] interactions to encapsulate the two components into polymer materials. For example, Xie et al. used poly(styrene/acrylamide) copolymer nanospheres to embed MNPs and QDs, the hydrophilic groups of the copolymer were inclined to locate on the outer surface of nanospheres. At the same time, numerous hydrophobic moieties were found in the interior, leading to the formation of hydrophobic cavities [64]. Both hydrophobic CdSe/ZnS QDs (3–6 nm) and Fe_2_O_3_ (5–20 nm) can be directly embedded into the mesoporous to form multimodal hybrid nanocomposites. The common polymer materials used in these studies include poly(styrene/acrylamide) copolymer nanospheres [64,72], poly(styrene-co-ethylene glycol dimethacrylate-co-methacrylic acid) beads [65], poly(lactic-co-glycolic acid) (PLGA) [76], poly(glycidyl methacrylate) [77], poly(lactide)tocopherol polyethylene glycol succinate [63], and chitosan-based polyelectrolyte complexes [67,78].

Template-based embedding techniques utilize biocompatible materials to adjust the performance of the obtained nanocomposites, intending to improve their stability and dispersibility, modify functional groups, and reduce toxicity. The carrier of a huge interior cavity provides opportunities for high payloads of dual components and easy manipulation of desirable properties by changing the proportion of different types of components. High loading with MNPs could enhance magnetic responsiveness intensity and separation speed under an external magnetic field, thereby minimizing the separation time in complicated food matrices. Moreover, the different emission QDs could be embedded into SiO_2_, enabling optical encoding of multiple food contaminants. The template embedding method to fabricate MNPs@QDs nanocomposites provides an ideal strategy for designing matrix tolerance and high-performance sensing probes.

### 2.3. Layer-by-Layer Assembly

LBL assembly integrates performed MNPs and QDs through non-covalent forces and chemical covalent bonds to form MNPs@QDs with a multi-layer self-assembled core-shell structure. The non-covalent interactions mainly involve electrostatic adsorption [79,80,81], hydrophobic [82], coordination [83], and biomolecule-assisted system [84,85]. The polyelectrolyte cationic polymers-mediated electrostatic adsorption is commonly used for preparing core-shell MNPs@QDs, such as the poly (allylamine hydrochloride)[79], poly (dimethyl diallyl ammonium chloride) [86], and polyethyleneimine (PEI) [80]. The negatively charged QDs were wrapped on the surface of positively charged PEI capped MNPs, while the luminescent intensity could be tuned by controlling the number of PEI layers to absorb different amounts of QDs [80,87,88] (Figure 3c). The amphiphilic poly(4-vinylpyrollidone) capped Fe_3_O_4_ nanoparticles can bind 1-dodecanethiol modified QDs via hydrophobic-hydrophobic interactions to form MNPs@QDs [82]. Alternatively, barnase-capped MNPs were tightly conjugated with barstar-capped QDs to form dual-functional MNPs@QDs via this protein-assisted noncovalent binding system [84], and the biotin-functionalized Fe_3_O_4_ conjugated with streptavidin-functionalized CdSe/ZnS QDs via the high-affinity streptavidin-biotin system [85]. The non-covalent interactions do not depend on complicated chemical reagents and synthesis, which provides a simple way to construct dual-functional MNPs@QDs nanocomposites. It is worth noting that the QDs may leak or drop from the linker-connected MNPs under certain conditions, which may affect the stability of the storage, coupling, and practical applications. Moreover, the emission intensity is inevitably affected by MNPs to reduce the PL QY, which may be attributed to non-radiative energy or charge transfer processes during the assembly.

Another approach for LBL assembly is based on covalent binding between MNPs and QDs. The strategy utilizes reactive functional groups such as carboxyl (-COOH), amino (-NH_2_), thiol, and siloxane groups to realize the connection of two components [89,90,91,92,93] (Figure 3c(ii)). NH_2_-Fe_3_O_4_@SiO_2_ is the most commonly used for coupling with COOH-QDs via the carbodiimide chemistry method [94,95]. Fe_3_O_4_ is easily modified with amino groups by the silanization treatment, and SiO_2_ wrapped on the surface of Fe_3_O_4_ minimized the quenching effect and provided functional groups for enabling chemical bonding with QDs while solving the problems of easy aggregation and increasing their stability. The l-cysteine-modified ZnS QDs with rich amino are also applied for coupling with COOH-functionalized Fe_3_O_4_ to form MNPs@QDs, but the coupling efficiency may be reduced when recognition elements were used directly conjugating with the outer layer of amino-QDs [90]. The thiol modified Fe_3_O_4_@SiO_2_ was used for binding QDs seeds on the surface. The carboxylic groups of the thiol ligands improved the water dispersity and surface functionality for further conjugation of bioactive molecules [96]. The 3-mercaptopropyltrimethoxysilane (APTES) capped ZnS QDs have trimethoxysilane groups, which can be easily connected via Si-O-Si bonds to create a SiO_2_ network and conglutinated together with Fe_3_O_4_@SiO_2_ in a nanosphere [92]. The covalent interaction provides a solid combination compared with non-covalent interactions. This approach increases the stability of nanocomposites and reduces the possibility of QDs leaking from the surface of the MNPs. The abundant functional groups of the dual components build a robust bridge for the construction of MNPs@QDs nanocomposites, and these active functional groups also provide diverse sites for the conjugation of specific recognition elements for convenient detection of multiple food contaminants. The LBL strategy to prepare MNPs@QDs nanocomposites has become popular due to its high simplicity, operability, and adaptability, satisfying the requirements of constructing rapid, sensitive multifunctional sensors for food contaminants analysis.

### 2.4. Microemulsion Technique

The microemulsion technique is a transparent or translucent, isotropic, thermodynamically stable system formed by water, organic solvents, MNPs, QDs, and surfactants in appropriate proportions. Chen et al. mixed hydrophobic QDs and MNPs with dodecyltrimethylammonium bromide (DTAB) to form an aqueous solution, and the mixture was then quickly poured into a poly(vinylpyrrolidone) (PVP) ethylene glycol (EG) solution. The obtained nanoparticles were 120 nm with a close-packed structure, MNPs preferentially became a magnetic core, QDs formed a fluorescent shell, and dipole-dipole interactions of MNPs and oleophobic interactions generated between MNPs and QDs promoted the structure formation [97]. A sol-gel process was introduced to encapsulate a thin silica shell on the surface of MNPs@QDs for improving biocompatibility and colloidal stability, and the obtained nanocomposites were successfully applied as a live cell tracer and dual-modal imaging probe [98].

Polymer beads for incorporating MNPs and QDs also attract considerable attention due to their simplicity and diversity. Guo et al. used trichloromethane containing octadecylamine-coated QDs (OC-QDs), oleic acid-modified MNPs (OA-MNPs), poly (methyl methacrylate) (PMMA), and poly (maleic anhydride-alt-1-octadecene) (PMAO) composites to form MNPs@QDs through an ultrasonic emulsification solvent evaporation process [99]. The fluorescence intensity was 226 times that of corresponding QDs, and saturation magnetization still retained 45.4% of the compared MNPs. The ultrasonic emulsification introduced herein could conveniently control the size of nanocomposites, rendering them more suitable for point of care testing applications. The amphiphilic (2-hydroxyl-3-dodecanoxyl) propylcarboxymethyl chitosan [100], PLGA [101], poly (styrene-co-maleic anhydride) [102] also act as carriers, in which MNPs and QDs dissolved in the organic phase are transferred into the aqueous phase through hydrophobic interaction. The general strategy of template embedment is depicted in Figure 3d. The reaction process involves using more organic solvents or surfactants, and incomplete evaporation and washing to remove these reagents may damage the dual properties of MNPs@QDs. Moreover, the materials yield via this method is low, which is not conducive to its wide applications in food contaminants’ rapid detection.

The microfluidic devices are also employed to simplify the process of producing standardized MNPs@QDs with uniform size and a controllable number of QDs within each particle. As depicted by Lan et al., the Fe_3_O_4_ and CdSe/ZnS QDs were respectively dispersed in the alginate solution within the corresponding inlet, and then co-flow was formed in the flow-focusing-channel [103]. Under the flow-focusing orifice, the Ca^2+^ in the oil phase was mixed with droplets, and many Janus droplets were produced by symmetric shearing, and droplets were solidified in the extension serpentine channel and collected at the outlet. The sodium alginate used herein provided an excellent carrier for cross-linked with Ca^2+^ to form a gel structure, and the COOH of the surface could be used for further biofunctionalization. Interestingly, Fe_3_O_4_ and QDs stayed in their respective hemispheres within a fairly symmetrical structure, which minimized the fluorescence quenching effect of MNPs and prevented the leakage of QDs. The microfluidic technology as a simple, convenient, and straightforward approach for producing MNPs@QDs with fantastic fluorescence and magnetism is of great potential, the dual properties of nanocomposites could be easily manipulated by adjusting the flow rate of liquid. The microfluidic-based microemulsion technique points the way for the production of standardized MNPs@QDs.

### 2.5. One-Pot Method

The one-pot method is to mix the precursors of MNPs and QDs in a vessel to complete the fabrication of MNPs@QDs in a single step. The hydrothermal method is a bottom-up strategy under high temperature and pressure and is frequently used for preparing MNPs@QDs nanocomposites in one step; their scheme illustration is presented in Figure 3e. In one study, Zhou et al. utilized graphite oxide (GO), cadmium chloride, ferric dichloride tetrahydrate, and sodium acetate as precursors and dispersed them in a DMSO solution to form a stable suspension. The mixture was then transferred into a Teflon-lined autoclave for a high-temperature reaction (180 °C for 12 h) to obtain nanocomposites [104]. The individual components were well distributed with no mutual interference. The high specific surface area and abundant negative charge of GO provided more nucleation sites for loading MNPs and QDs. The assembled nanocomposites exhibited favorable magnetism intensity (44.85 emu/g) and high loading efficiency (0.98 mg/mg) for doxorubicin.

In addition to QDs, carbon quantum dots (CQDs) were also employed to fabricate MNPs@QDs nanocomposites. There are two apparent merits of CQDs to prepare nanocomposites: (i) the inherent advantages of CQDs, such as low cost, low toxicity, high surface area, abundant surface groups, favorable optical properties; (ii) the electrostatic repulsion generated by CQDs, which provides excellent colloidal stability for Fe_3_O_4_. Maleki et al. added FeCl_3_·6H_2_O, ethylenediamine, and citric acid into deionized water and poured it into a Teflon-lined autoclave for heating at 200 °C for 5 h, the MNPs@QDs was synthesized, and its magnetism intensity reached 62.0 emu/g [105]. The CQDs derived from a onefold carbon source suffer a low QY, and heteroatom doping plays a crucial role in regulating the fluorescent intensity of CQD. Nitrogen-doping (N-doping) is a common method to improve QY. In the Liu et al. research, Poly-γ-glutamic acid (γ-PGA) was utilized as both a carbon and nitrogen source at the same time, and the precursors experienced heating and stirring, pH control, aging, and high-temperature forming Fe_3_O_4_@CQDs [106]. The QY and magnetism intensity of resulting nanocomposites were 21.6% and 62 emu/g, respectively. The superior characteristics of high QY, good dispersity, excellent colloidal stability, tunable fluorescence, high QY, and strong magnetism make them an advanced probe for triple-modal tumor imaging. In another study, ferric ammonium citrate acted as an iron precursor and carbon source, and triethylenetetramine (TETA) acted as nitrogen source and reducing agent, followed by high-temperature treatment to obtain Fe_3_O_4_@CQDs in one convenient step [107]. TETA effectively improved the adhesion of CQDs and Fe_3_O_4_ and gained better crystallinity. The QY of Fe_3_O_4_@CQDs drastically decreased to 4.6% compared with TETA-CQDs (53%). The static and dynamic fluorescence quenching of CQDs and IFE generated by MNPs conspire to cause this phenomenon. Although the one-pot method provides a rapid, simple, and economic strategy for fabricating bifunctional nanocomposites, the selection of suitable precursors is directly related to the magnetic and fluorescent properties of MNPs@QDs nanocomposites. Impurities are inevitably generated during the reaction period, which affects the separation and purification of products, and cannot achieve precise control of fluorescence and magnetic properties.

Doping transition metal ions or lanthanides into a crystalline lattice of QDs is another strategy for the one-pot preparation of MNPs@QDs. The transition metal ions and lanthanides such as Mn^2+^ [108,109], Eu^3+^ [110], Gd^3+^ [111,112], and Ln^3+^ [113] are used for preparing doped MNPs@QDs. The doped materials are mainly concentrated in biomedical applications and are seldom involved in the rapid detection of food safety. The magnetic intensity of doped materials may be insufficient for the separation and enrichment of complex sample matrix.

## 3. Applications to Food Contaminants Analysis

Currently, MNPs-based rapid sample separation and QDs-based fluorescent labeling are extensively used in food contaminant analysis [114]. The MNPs@QDs simultaneously integrate the functions of separation and labeling, which can quickly enrich and separate the targets from the complicated food matrices under the action of the external magnetic field and realize the “fluorescence switch” quantitative analysis of the target analytes at the same time, simplifying the sample pretreatment, reducing the loss of the analyte, dramatically shortening the detection time, and improving the detection efficiency. The bifunctional MNPs@QDs with excellent performances have been successfully applied to detect food contaminants, including metal ions, foodborne pathogens, toxins, pesticides, antibiotics, and illegal additives. In this section, we summarize in detail the application of MNPs@QDs-based sensors for the detection of food contaminants in food samples.

### 3.1. Metal Ions

Presently, toxic metal ions (Cu^2+^, Hg^2+^, Pb^2+^) have attracted attention due to their significant side effect. These metal ions are an essential risk factor causing water and environmental pollution. Even if trace levels of metal ions enter humans through the food chain, the health harm to human reproduction, nerve, and cardiovascular systems will produce [115]. Therefore, a rapid, sensitive, efficient, and low-cost method for detecting metal ions is significant to ensure food safety.

**Figure 4:**
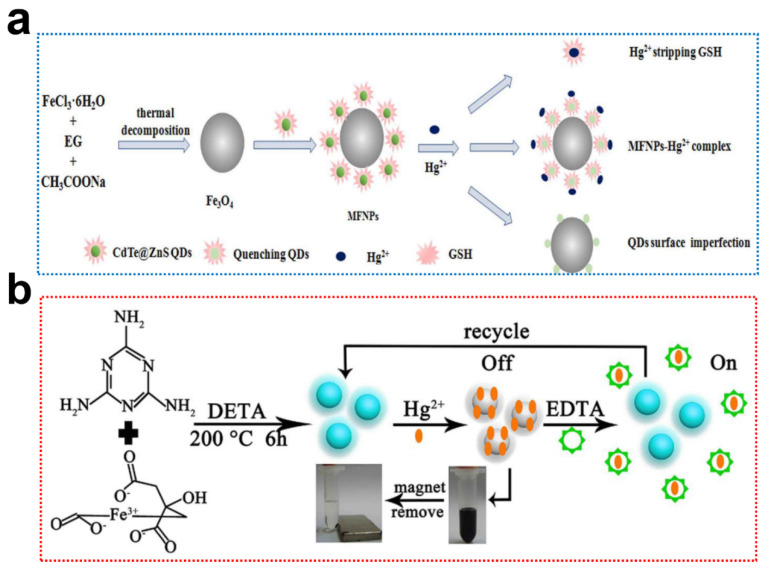
(**a**) Schematic illustration of MNPs@QDs-based nanosensor for Hg^2+^ detection. Reproduced with permission from [122]. Copyright Ceramics International, 2018; (**b**) Schematic illustration synthesis strategy of Fe_3_O_4_@CQDs and utility in sensing and removal of Hg^2+^. Reproduced with permission from [127]. Copyright Food Chemistry, 2021.

The specific surface and structural properties of chitosan (CS) provide excellent chelating ability for metal ions [116,117,118,119,120]. Li et al. designed an adsorbent-chemosensor based on Fe_3_O_4_@CS@CQDs for selective detection of Hg^2+^ in water [121]. The introduction of CS/CQDs significantly increased the absorption capacity with a maximum monolayer adsorption capacity of 110. 62 mg/g, while the surface defect of QDs promoted the migration and coupling with Hg^2+^, resulting in fluorescence quenching, achieving a linear range of 0–4 mM with a limit of detection (LOD) of 12.43 nM. The carboxymethyl chitosan (CMCS) microspheres were used for encapsulating MNPs and QDs to fabricate bifunctional MNPs@QDs. The abundant active functional groups of CMCS and electrostatic interaction promoted the binding of Hg^2+^ [122]. The proposed sensor achieved a linear range of 0.3–5 μM with a LOD of 0.091 μM (Figure 4a). The abundant amino, carboxyl, and thiol groups of glutathione (GSH) provided a favorable carrier for highly efficient absorption [123], a GSH modified MNPs@QDs nanosensor for simultaneous detection and removal of Cu^2+^ was well developed [124]. The method features a linear relationship varying from 5 to 30 μM with a LOD of 0.2 μM. In another study, the di-2-picolylamine/proline co-modified Fe_3_O_4_@ZnS nanocomposites were used for the removal and detection of Cu^2+^ [125]. The maximum adsorption capacity of the nanocomposites reached 517.9 mg/g, and the nanocomposites could quantificationally detect Cu^2+^ in the range of 6–20 μM. The biocompatible calcium carbonate (CaCO_3_) crystals also acted as a carrier for encapsulating AgInS_2_/ZnS ternary QDs and Fe_3_O_4_ to prepare CaCO_3_-Fe_3_O_4_@AgInS_2_/ZnS fluorescent sensor for simultaneous detection of three metal ions [126]. The positively charged Co^2+^, Ni^2+^, and Pb^2+^ interacted with negatively charged AgInS_2_/ZnS QDs to produce a fluorescence quenching, and LODs were 10 nM for Co^2+^, and 100 nM for both Ni^2+^ and Pb^2+^. Although good sensitivity is provided by proposed sensors, the deficiency of selectivity reduces its practicality.

Directly using MNPs@QDs nanocomposites without the assistance of a linker is an attractive option to simplify procedures further and improve the adaptability of MNPs@QDs for metal ion detection. Xie and Co-workers utilized the one-pot method to prepare Fe_3_O_4_@CQDs and simultaneously detect and remove Hg^2+^ in contaminated water samples [127]. The Fe_3_O_4_@CQDs exhibited a strong blue fluorescent emission at the band of 435 nm; the QY was calculated to be 58%. The abundant active functional groups of CQDs promoted the interactions with Hg^2+^ and accelerated non-radiative recombination by an effective electron transfer process. The method manifested a linear response in the range of 0.003–0.01 μM with a LOD of 0.3 nM (Figure 4b), and their practicality was verified in spiked lake water, tap water, and drinks with average recoveries of 96.5–108.8% with an RSD lower than 6.0%. Dong et al. conjugated CQDs with Fe_3_O_4_@SiO_2_-NH_2_ nanocomposites via amine-carbonyl reactions and designed a fluorescent sensor for Cu^2+^ detection [89]. The LOD of Cu^2+^ was calculated to be 0.16 μM with a linear range of 0–80 μM. The graphene quantum dots (GQDs) with large surface areas, abundant active groups, and π–π conjugated networks have a better affinity toward metal ions [128]. Alvand and Shemirani fabricated Fe_3_O_4_@SiO_2_@GQDs nanocomposites and utilized them for dual functional detection and the removal of Hg^2+^ in water samples [129]. The nanocomposites exhibited a fast intake of Hg^2+^ within 1.5 min, and the maximum adsorption capacity was 68.03 mg/g. The linear range of the sensor covered 0.1–70 μM, with a LOD of 30 nM. Wang et al. encapsulated the CdTe QDs and Rhodamine 6G (Rh6G) into mesoporous SiO_2_ to fabricate multifunctional inorganic-organic nanocomposites for simultaneous removal and detection of Hg^2+^ [130]. The maximal adsorption capacity of nanocomposites reached 17.7 mg/g and could be reused 8-time recycling. The proposed sensor for the ratiometric analysis of Hg^2+^ ranged from 7–900 nM with a LOD of 2.5 nM and had satisfactory recoveries of 97.6–102.3% in deionized water and tap water. The elements doped MNPs@QDs were also used for metal ions sensing [131,132]. The one-step hydrothermal method for preparing Mn-doped CQDs was simultaneously applied as a biosensing probe for Fe^3+^ and magnetic resonance imaging [133]. The Mn-CQDs presented a bright, strong yellow-green fluorescence at the emission band of 464 nm, and QY was 13%. The oxygen-containing functional groups on the surface of MnCQDs promoted the coordination with Fe^3+^ and resulted in a fluorescence quenching. The fluorescence intensity was inversely proportional to the concentration of Fe^3+^, with a linear range of 0–1.2 μM and LOD of 0.22 μM. Wu et al. synthesized tetragonal chalcopyrite crystalline structured ternary CuFeS_2_ QDs for simultaneous sensing Cu^2+^ and Fe^3+^ [134]. Both Cu^2+^ and Fe^3+^ coordinated with CuFeS_2_ QDs to produce an aggregation effect and cause obvious fluorescence quenching. The method showed LODs for Cu^2+^ and Fe^3+^ lower to 1.98 and 2.15 μM, respectively.

The applications of MNPs@QDs-based sensors for detecting metal ions are depicted in Table 1. Sensors for metal ion detection based on MNPs@QDs nanocomposites are rapidly developing and have great potential for metal ion adsorption, detection, and removal, which will provide a simple, rapid, accurate, and reliable tool for tracing metal ions in food safety monitoring.

### 3.2. Foodborne Pathogens

Foodborne pathogens, including *Escherichia coli* O157:H7 (*E. coli* O157:H7), *Salmonellas*, *Pseudomonas aeruginosa*, etc., are among the leading causes of common foodborne disease outbreaks worldwide [135]. The human consumption of food contaminated with pathogens could result in severe vomiting and diarrhea [136]. Therefore, on-site detection of foodborne pathogens is of greatly signifcant for ensuring food hygiene and promoting public health [137].

**Figure 5:**
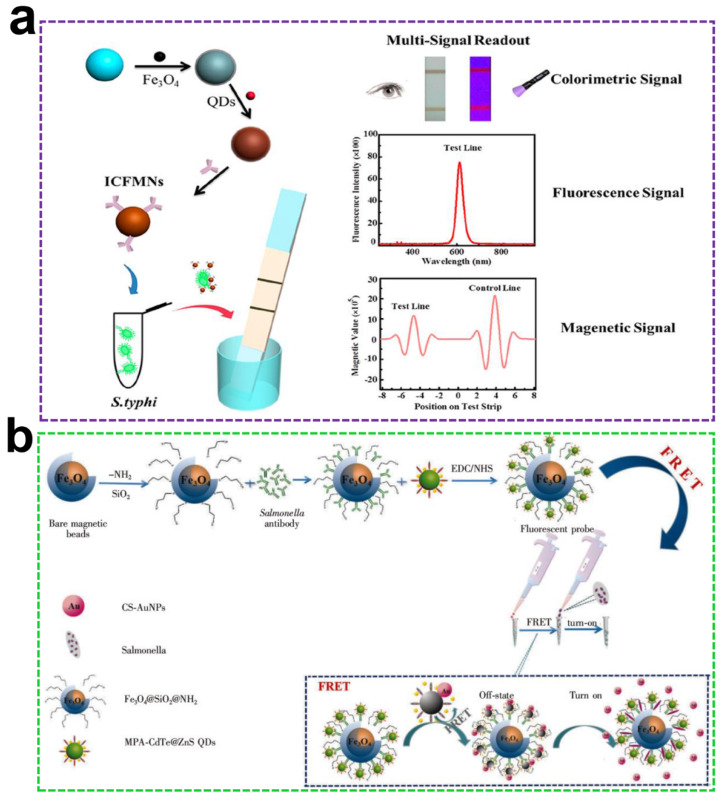
(**a**) Schematic diagram for triple signal output detection of *S. typhi* by using MNP@QDs-based-LFIA. Reproduced with permission from [140]. Copyright Analytical Chemistry, 2019; (**b**) Schematic illustration of an off-on fluorescent probe for rapid detection of *S. typhi* based on FRET effect. Reproduced with permission from [148]. Copyright Chinese Journal of Analytical Chemistry, 2019.

Lateral flow immunoassay (LFIA), characterized by the merits of rapid, low-cost, and on-site detection, has been widely used for many samples screening [138]. Huang et al. constructed a sandwich LFIA immunosensor to detect *E. coli* O157:H7 using a bifunctional MNPs@QDs probe [139]. The monoclonal antibody (mAb) was conjugated with MNPs@QDs to form a detection immunoprobe. The rabbit polyclonal antibody (pAb) was immobilized on the test (T) line to capture free *E. coli* O157:H7, and the high specific antibody guaranteed the selectivity of the method. The fluorescence intensity was positively correlated with the number of *E. coli* O157:H7 linear range from 2.5 × 10^2^ CFU/mL to 5 × 10^5^ CFU/mL, and LOD was 2.39 × 10^2^ CFU/mL. The high sensitivity benefit from 6.57–10 times magnetic enrichment and the removal of matrix interference of milk, while the omitted elution and incubation process avoid the loss of target and improve detection efficiency. The separation and enrichment, tripe mode signal output, and two formats of quantitation were integrated into one multimodal assay platform to detect *Salmonella typhimurium* (*S. typhi*) [140]. Figure 5a depicts the test principles of the bifunctional nanocomposites-based triple mode sensing platform. The visual LODs (vLODs) of the color and fluorescence signal were 1.88 × 10^4^ CFU/mL and 3.75 × 10^3^ CFU/mL, respectively. A good linear relationship was achieved in the range of 1.88 × 10^4^–1.88 × 10^7^ CFU/mL with a LOD of 3.5 × 10^3^ CFU/mL for both fluorescence and magnetic signal. The 2–4 orders of magnitude improvement were observed with gold nanoparticles (AuNPs)-LFIA. Ghasemi et al. conjugated NH_2_-Fe_3_O_4_@SiO_2_ with COOH-QDs to form core-shell MNPs@QDs, and high specific mAb was immobilized on the surface of nanocomposites for pre-enrichment of *Streptococcus agalactiae* (*S. agalactiae*) from milk [141]. The immune-MNPs@QDs captured with *S. agalactiae* to form a large-grained immunocomplex and could not pass the 60 nm size pore of filters, and fluorescence intensity was directly proportional to the number of *S. agalactiae*. The LODs for *S. agalactiae* detection in PBS and milk were 10 and 100 CFU/mL, respectively. The difference could be attributed to fat, protein, and minerals in milk reduced the capture efficiency.

Aptamers are short-chain oligonucleotides (DNA or RNA) that can recognize and bind targets with excellent affinity with the characteristics of chemical stability, low immunogenicity, and automated synthesis. The aptamer-based sensors have been extensively used for monitoring food safety [142,143]. The aptamer was combined with MNPs@QDs to construct a fluorescent sensor for highly sensitive detection of *S. typhi* in vegetable samples [144]. The aptamers-modified MNPs@QDs produced an aggregation effect upon the capture of *S. typhi* cells resulting in fluorescence quenching and were separated and redispersed for fluorescence detection. The LODs of the sensor in the fresh-cut vegetable washing solution and lettuce sample were 1 × 10^2^ and 1.38 × 10^2^ CFU/mL, respectively, and satisfactory recoveries in these two types of samples were achieved between 94.0% and 107.5%. Lin et al. used APTES, γ-Fe_2_O_3_, and QDs as functional components to assemble MNPs@QDs, and by adjusting the number of MNPs to obtain two MNPs@QDs with various magnetic responsiveness [145]. These two MNPs@QDs respectively conjugated *E. coli* O157:H7 and *S. typhi* aptamer with the assistance of the streptavidin-biotin system. The two MNPs@QDs immunocomplexes present differentiated magnetic responsiveness under the same external magnetic field, and the immunocomplexes were respectively separated at different immunoreaction times for fluorescence quantitative analysis. The *E. coli* O157:H7 detection had a linear range of 40–10^8^ CFU/mL with a LOD of 16 CFU/mL, and the *S. typhi* had a linear range of 63–10^8^ CFU/mL with a LOD of 25 CFU/mL. The mean recoveries of *E. coli* O157:H7 and *S. typhi* in milk samples were 87.6–97.7% and 84.9–95.9%, respectively, and the RSD of both pathogens were in the range of 1.1–5.9%. The high recoveries of this method could be attributed to the following three points: (i) high affinity and specificity of aptamer-modified nanoprobes for the capture of pathogens; (ii) high luminescent intensity improved the detection sensitivity; (iii) variable magnetic response of nanoprobes effectively separated each pathogen at different time points. The high sensitivity and wide quantitative range achieved in this study could satisfy the requirements of different levels of the same pathogens in various regions.

Several works constructed fluorescent nanoswitch control for pathogens detection based on MNPs@QDs nanocomposites. Ahmadian-Fard-Fini et al. used grapefruit, lemon, and turmeric extracts to prepare the blue emissive CQDs with QY of 20%, and they were also utilized as capping agents to prepare Fe_3_O_4_@CQDs nanocomposite [146]. The fluorescence of Fe_3_O_4_@CQDs presents inversely proportional to the number of *E. coli* in the range of 0–10^9^ CFU/mL. A “turn-off” fluorescence response strategy herein was introduced for pathogens detection. A similar approach was also employed for *Pseudomonas aeruginosa* detection by using Mg^2+^ [132] and Ni^2+^ [131] doped MNPs@CQDs. Wang et al. used thiolated MNPs@SiO_2_ linked with thioglycolic acid-modified CdTe/CdS QDs to form bifunctional nanocomposites and introduced a “turn-on” strategy for *Alicyclobacillus* spp. detection [147]. In this study, both synergistic effect and electrostatic interaction promoted the fluorescence enhancement during the process of immuno-MNPs@QDs capture *Alicyclobacillus* spp. The minimum LOD was 10^4^ CFU/mL, and the whole testing process was completed within 90 min. Cui et al. utilized a “turn-off-on” strategy for the detection of *S. typhi* based on the fluorescence resonance energy transfer (FRET) effect between QDs and AuNPs [148]. The absorption spectrum of AuNPs overlapped with the emission spectrum of QDs, and the distance between AuNPs and QDs was reduced to less than 10 nm; satisfaction with these two factors triggered FRET to quench the fluorescence of QDs. The addition of bacterial cells increased the distance and diminished the FRET effect, the LOD was lower to 1.7× 10^2^ CFU/mL, and the assay could be completed within 2 h (Figure 5b).

The performances of the MNPs@QDs-based sensors for foodborne pathogens detection are summarized in Table 2. The LFIA and homogenous fluorescent methods are the most widely used detection forms for foodborne pathogens determination. Combining high-affinity recognition elements with MNPs@QDs makes them a powerful pretreatment tool for capturing target pathogens, while high-performance detection probes are used for constructing a fluorescent nanoswitch for sensing foodborne pathogens. The pretreatment and detection were simultaneously integrated into one sensing platform, which will bring significant improvement in foodborne pathogens detection.

### 3.3. Toxins

**Figure 6:**
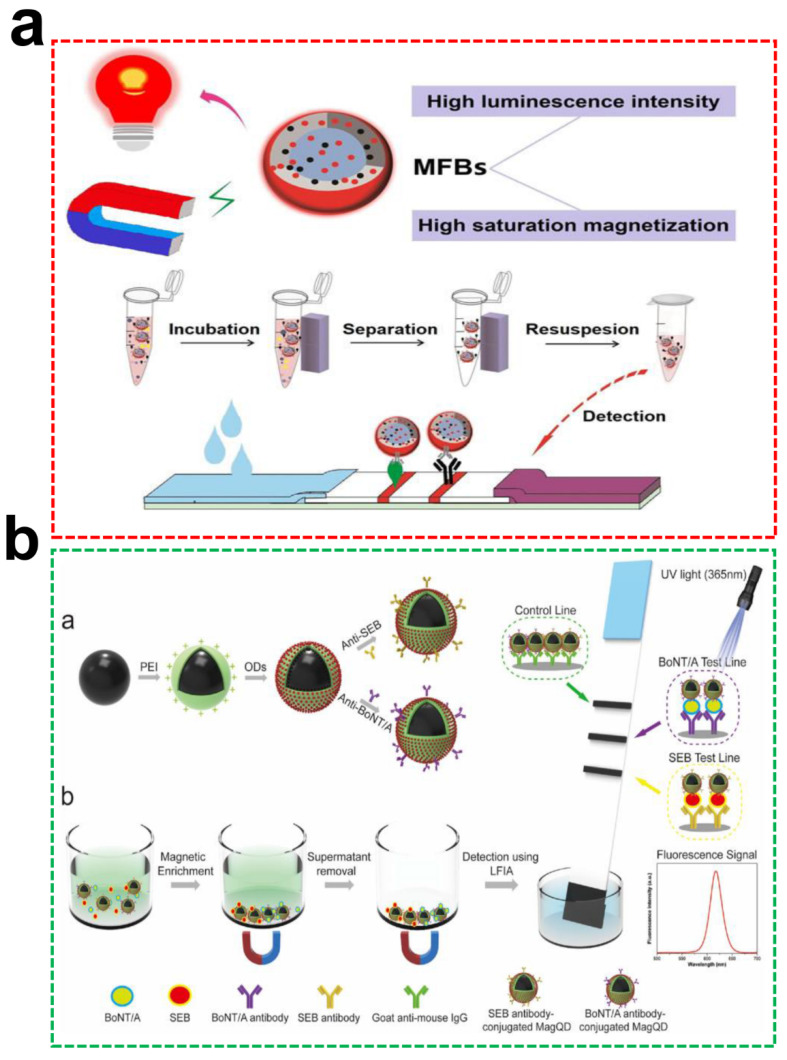
(**a**) Schematic illustration of bifunctional MNPs@QDs-based LFIA for AFB_1_ detection in dark soy sauce. Reproduced with permission from [99]. Copyright Analytical Chemistry, 2019; (**b**) The insert (**a**) represent scheme of antibody-modified MNPs@QDs preparation and (**b**) test principles of MNPs@QDs-based two-channel LFIA strip for simultaneous and sensitive detection of two protein toxins. Reproduced with permission from [152]. Copyright Biosensors and Bioelectronics, 2019.

Mycotoxins are metabolites produced by fungi such as molds and usually exist in moldy food and feed. Even trace levels of mycotoxins intake by human beings can also cause diseases with kidney disease, liver disease, and cancer, posing a serious health threat to people and animals [149]. Therefore, a rapid, simple, and highly sensitive on-site detection method prioritizes ensuring human health and preventing contamination from related products. Among them, aflatoxin is a naturally occurring, highly-toxic carcinogen classified by the World Health Organization, which mainly exists in grain, oil, and their related products [150]. Guo et al. utilized the microemulsion technique to prepare core-shell bifunctional MNPs@QDs with favorable fluorescence and magnetism [99]. The surface of OA-modified QDs contained an abundant COOH group for coupling with high-affinity aflatoxin B_1_ mAb through carbodiimide chemistry. It is noteworthy that the fluorescence intensities were not wholly recovered even after 1000-fold sample dilution in direct detection mode. In enrichment detection mode, the antibody-labeled MNPs@QDs were used for enriching AFB_1_ molecules and removing pigments in dark soy sauce under an external magnet; just a 6-fold dilution was enough to eliminate the matrix interference with the 167-fold improvement compared with direct detection mode, which indirectly improved the sensitivity of the method. Under optimal conditions, the proposed method achieved a linear range from 0 to 150 pg/mL (R^2^ = 0.9931), with a IC_50_ of 27 ± 3 pg/mL. The LODs were 3 and 51 pg/mL in soy sauce extract and real dark sauce, respectively. The mean recoveries of AFB_1_ in black soy sauce were all higher than 89%, with a CV lower than 12%. The MNPs@QDs-based LFIA realized sample-to-answer within 45 min, and their test process is shown in Figure 6a.

Bacteria secrete a wide variety of protein toxins, such as cholera toxin, botulinum neurotoxins (BoNT), staphylococcal enterotoxins (SE), and Shiga toxin, which can cause serious foodborne diseases and substantial economic losses [151]. Given the extremely low concentration (lower than 1 ng/mL) and wide variety of these toxins, sensitive and multi-channel methods need to be developed to identify them from complex samples. A PEI-mediated Fe_3_O_4_@CdSe/ZnS QDs were well fabricated as a novel advanced bifunctional probe for simultaneous enrichment and detection of BoNT/A and SEB with high sensitivity [152]. The proposed method provides a dual signal readout, and the vLOD of the fluorescence signal for both target toxins was 10 pg/mL and lower than that of the brown color signal (1 ng/mL). The LODs for BoNT/A and SEB were calculated to be 2.52 pg/mL and 2.86 pg/mL, respectively, which achieved a significant improvement of approximately 396-fold for BoNT/A and 349-fold for SEB compared with color results. The MNPs@QDs based two-channel LFIA could complete the entire process in 30 min, including 20 min of magnetic separation pretreatment and 10 min of chromatography (Figure 6b). The milk and juice were employed as actual samples for verifying practicability, and recoveries were in the range of 78.8–98.0%, with an RSD lower than 10.3%.

The existing methods for detection of toxins based on MNPs@QDs are listed in Table 3. The currently reported bifunctional MNPs@QDs-based LFIA for detecting toxins are scarce and mainly focused on the form of LFIA. More applications and forms need to be discovered in subsequent research.

### 3.4. Pesticides, Antibiotics and Illegal Additives

Pesticides, including insecticides, herbicides, etc., are extensively used in agricultural production [153]. Unreasonable use will lead to its gradual accumulation in soil, water, and agricultural products, and the risk of human exposure to pesticides will also increase accordingly. Rapid and accurate analysis of these residues is an essential topic in food safety monitoring, and novel nanomaterials based on MNPs@QDs nanocomposites provide an alternative method for pesticide residue analysis. In the face of complicated food matrices, the MIP with specific recognition is usually selected for preconcentrating and subsequent detection. Embedding MIPs to form MNPs@QDs as trifunctional nanocomposites for rapid separation, specific recognition, and fluorescence detection is an interesting strategy. Li and co-workers designed Fe_3_O_4_@SiO_2_@CdTe QDs-MIPs nanocomposites for extracting and tracing the trichlorfon in vegetable samples with a LOD of 30 μg/kg [154]. Good recoveries were also observed in spiked cucumber and cauliflower samples, with average recoveries from 78.7% to 96.6%. In another study, Zhu et al. utilized alkoxysilane groups of silane-modified CQDs copolymerized with Fe_3_O_4_@SiO_2_ and constructed a novel sensor for sensitive detection of 4-nitrophenol (4-NP) [155]. The proposed sensor for 4-NP detection with a good linear range from 0.08–10 μM, and LOD was 23.45 nM. Flesh and head samples from fished recovered from the water ranged from 93.2 to 102.2%, with an RSD lower than 5.0%. The five remove-rebinding cycles remained stable fluorescence response with low RSDs, confirming its practical and inexpensive applications in real samples. Moreover, dual recognition derived from imprinted recognition based on hydrogen bond between 4-NP and APTES and fluorescence identification based on nitro dynamic quenching effect is the primary factor for strengthening specificity and sensitivity. Hu et al. employed Mn-doped ZnS QDs as a fluorescent core, TEOS as a cross-linker, assembly Fe_3_O_4_@SiO_2_ to form MIPs modified MNPs@QDs [156]. This nanocomposite presented a good fluorescence response to N-Nitrosodiphenylamine. The linear range was 0–120 μM, and LOD was lowered to 0.69 μM. The inner effect generated by the overlapping of UV-Vs absorption of N-Nitrosodiphenylamine with the fluorescence excitation spectrum of MIPs-modified MNPs@QDs may be the fluorescence quenching mechanism.

**Figure 7:**
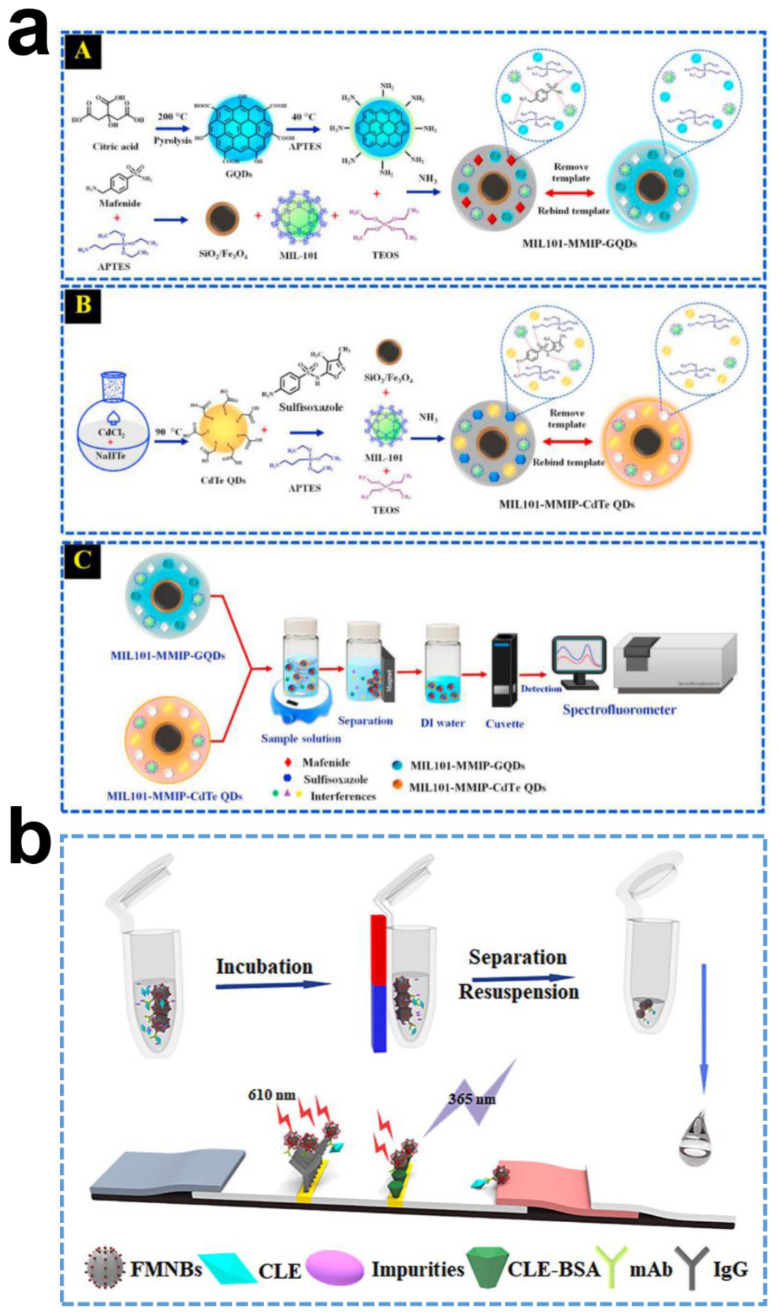
(**a**) Scheme shows the fabrication procedures of (**A**) MIL101-MMIPs-GQDs probe and (**B**) MIL101-MMIP-CdTe QDs probe, (**C**) and their applications in simultaneous detection of mafenide and sulfsoxazole using the two nanohybrid optosensing probe. Reproduced with permission from [163]. 2021, Copyright Microchemical Journal; (**b**) Schematic illustration of bifunctional LFIA sensor integrate the process of sample pretreatment and rapid detection of clenbuterol in swine urine. Reproduced with permission from [166]. 2019, Copyright Journal of Agricultural Food Chemistry.

Antibiotics have been extensively used for treating bacterial infections in humans and animals and used as feed additives to promote animal growth [157]. Animal-derived foods containing antibiotic residues not only directly produce toxic effects after entering the human body through the food chain but also indirectly increase the risk of bacterial resistance transmission. It is necessary to trace these hazardous substances from the source [158]. The NH_2_-Fe_3_O_4_ were conjugated with thiolated QDs via covalent binding to form MNPs@QDs nanocomposites and were used for sensing tetracycline hydrochloride [159]. The fluorescence intensity gradually decreased with the addition of tetracycline hydrochloride, and the linear range was 10–700 nM with a LOD of 1.2 nM; the quenching mechanism was probable dynamic quenching. Dual functional components combined with mesoporous Fe_3_O_4_@SiO_2_ contribute to forming more recognition cavities in MIPs. This will achieve better recognition efficiency. Chen et al. developed a novel room-temperature phosphorescence MNPs@QDs-MIPs probe for tracing norfloxacin residue in milk and fish [160]. The MNPs@QDs-MIPs realized a highly specific fluorescence response to norfloxacin, and linearity ranged from 1 to 90 μg/mL with a LOD of 0.8 μg/mL. Its practicability was verified in the fish and milk samples with recoveries of 90.9–111.5%. The whole detection procedure was finished within 40 min. The efficient recognition of norfloxacin by nanocomposites with a large number of imprinting and binding sites directly improved the assay sensitivity, the dynamic quenching generated by photoinduced electron transfer is the main quenching mechanism. A similar strategy was also employed for detecting ceftazidime in the milk samples with a LOD of 0.05 μg/mL [161]. The binding affinity between target analytes and MNPs@QDs could be improved by introducing high-affinity materials with large specific surface areas into MIPs. Porous graphene, GQDs, and MNPs were integrated with MIPs to form a nanohybrid sensing probe and constructed an optosensor for levofloxacin detection [162]. The porous graphene and GQDs jointly promote the transfer of levofloxacin to recognition sites, inducing a fast response to levofloxacin through hydrogen bonding and π-π interaction. The quantitative linear range from 0.1 to 25 μg/mL with a LOD of 0.03 μg/mL. Cefoperazone has also been identified in another study via this strategy [163]. The metal-organic framework was employed as an affinity material with the merits of having a large surface area, high porosity, and tunable pore size. The GQDs with an emission band of 435 nm and CdTe QDs with an emission band of 572 nm were respectively fabricated MIL-101-MNPs@GQDs and MIL-101-MNPs@CdTe QDs, which could respectively detect mafenide, and sulfisoxazole in one sample [164]. The proposed optosensor showed good linearity for mafenide and sulfisoxazole detection in milk from 0.1–25 μg/mL, and LOD of both was 0.1 μg/mL. The average recoveries of this method in milk were 80.4–97.9% and 82.1–97.4% for mafenide and sulfisoxazole, respectively, with the RSD ranging from 0.3–4.6%. The study firstly realized the simultaneous detection of two analytes based on various MIL-101-MNPs@QDs with the same excitation (Figure 7a).

In the modern food industry, food additives play a crucial role in ensuring flavor, improving quality, and extending the shelf life of food. However, some illegal additives have raised new food safety issues and public concerns regarding food safety. Researchers are devoted to developing new sensors to solve these problems [165]. Clenbuterol, also commonly known as “lean meat powder,” has been rigorously banned as a feed additive in China and other countries due to its significant side effects. Huang et al. developed a sensitive and matrix-tolerant LFIA based on MNPs@QDs for on-site detection of clenbuterol [166]. The LBL strategy was used to synthesize Fe_3_O_4_@SiO_2_@CdSe/ZnS QDs with a typical core@shell@satellite structure. The fluorescence characteristics were not altered in the forming of nanocomposites, and the nanospheres could be recycled entirely in 3 min under magnetic separation, demonstrating the superior fluorescence and magnetic properties of Fe_3_O_4_@SiO_2_@CdSe/ZnS QDs. The complex components of urine could seriously interfere with the accuracy of LFIA results. The AuNPs-based LFIA presents a significant difference between urine and PBS, and the bifunctional MNPs@QDs were not affected by the urine matrix. In addition, the LODs of MNPs@QDs-based LFIA were 0.16 and 0.22 ng/mL in PBS and swine urine, respectively, which was four times lower than AuNPs-based LFIA. Recoveries for swine urine samples (0.5–2.0 ng/mL) were between 79.1 and 108.9%, with a CV in the range of 3.4–10.3%. The proposed method innovatively integrated magnetism and fluorescence in one probe to allow simultaneous enrichment and detection. This avoided the loss of analyte and improved sensitivity (Figure 7b). The microemulsion technique was utilized to fabricate CdTe QDs/nano-Fe_3_O_4_@MIPs for sensitive and selective detection of malachite green in fish [167]. The nanocomposites exhibited a fast absorption equilibrium of malachite green within 5 min and achieved a LOD of 0.014 μM. The recoveries of malachite green in fish samples ranged from 102.7% to 108.6%. In another study, polydopamine-modified Fe_3_O_4_@SiO_2_ to dop QDs through self-polymerization to form a chemiluminescent (ECL) probe and applied for the ultrasensitive detection of bisphenol A [168]. Free bisphenol A in the sample entered the recognition cavities and occupied the binding sites of MIPs, the strong ECL emission of QDs was blocked. In accordance with the ECL intensity, the detection performance of the sensor with a wide linear range from 1 nM to 0.1 mM, and LOD was lower to 0.34 nM.

As reviewed thus far, some progress has been achieved in detecting pesticides, antibiotics, and illegal additives based on bifunctional MNPs@QDs nanocomposites, and their detection performances are listed in Table 4. The combination of novel MNPs@QDs nanocomposites with high-affinity recognition elements is an attractive strategy to solve the problems of wide varieties, low concentration levels, and complex samples matrix in the detection of these contaminants.

## 4. Conclusions and Perspectives

The development of high-throughput, on-site, and portable analysis techniques has become a research hotspot for identifying food contaminants. The MNPs@QDs-based novel sensors have been extensively applied for food contaminants detection with superior detection performances. This review briefly overviews the preparation methods of MNPs@QDs nanocomposites and their applications in food contaminants detection. Despite great efforts that have been exerted by scientists to develop simple preparation methods to fabricate MNPs@QDs nanocomposites with excellent magnetic/fluorescent performances, the preparation process reported in the existing studies is cumbersome, and their stability and biocompatibility are usually unsatisfactory. The introduction of MNPs will inevitably cause the fluorescence quenching of QDs, which confirm that the development of MNPs@QDs nanocomposites with good adaptability still faces tremendous challenges. The integration of sample pretreatment and rapid detection in one sensing platform is attractive, but the complexity of sample pretreatment is a universal challenge for sensing platforms. The lack of standardized pretreatment protocols of MNPs@QDs nanocomposites for the inherent characteristics of specific food types may limit the development of sensors. More detailed information is required to comprehend the process of targets separation and recovery from the food matrix. Moreover, the analytical modes focused on several limited forms, which could not meet the diverse requirements in the face of various food matrices under different usage scenarios.

Despite these limitations, sensing platforms based on MNPs@QDs nanocomposites have great potential for determining contaminants in complicated food matrices and are rapidly developing. Herein, we propose several vital points for the future development of MNPs@QDs nanocomposites.

1. Pointing towards requirements of strong matrix tolerance and high QY, a universal method should be developed to simplify the preparation process and obtain a multifunctional MNPs@QDs probe.
2. In-depth exploration of the adsorption, enrichment, and separation procedures between MNPs@QDs and food contaminants, to provide a theoretical basis for tailoring the appropriate pretreatment protocols with various characteristics of food samples.
3. Combining high affinity, specific and stable recognition elements (such as MIPs, aptamers, and nanobodies) to construct rapid, sensitive, and high-throughput sensing platforms for food contaminants detections through different analytical forms.
4. Miniaturized and portable equipment integrated with sensing platforms for immediate on-site detection to confront various food safety incidents. The smartphones and microfluidic technologies that belong to smart manufacturing also provide a future development direction for analysis devices.

Simplifying the pretreatment process, improving the detection efficiency, and enhancing the anti-interference ability of the method are the future development directions in food contaminants analysis. Our goal is to stimulate broader interest in developing MNPs@QDs-based sensing platforms and improve their applications in food safety monitoring. We believe that emerging new insights and solutions generated by the collaborative endeavors of scientists in various fields will greatly accelerate the development of this area.

## Figures and Tables

**Table 1:** Detection performances of MNPs@QDs-based sensors for metal ions.

Analytes	Nanocomposites	Synthetic Strategy	Samples	LOD	Linear Range	Remarks	Reference
Cu^2+^	Fe_3_O_4_@SiO_2_-NH_2_/CQDs	LBL assembly	Water	0.16 μM	0–80 μM	Enhanced selectivity and sensitivity; eco-friendly	[89]
Cu^2+^	Fe_3_O_4_-CS@CdSeS QDs	LBL assembly	Tap/spring water	0.022 ng/mL	0.073–80 ng/mL	Simultaneous removal and optical detection; high saturation adsorption capacity; good sensitivity and selectivity	[117]
Cu^2+^	Fe_3_O_4_@OCMC@CQDs	One-pot method	Water	0.56 μM	0.01–200 μM	Enhanced sensitivity and selectivity	[118]
Cu^2+^	Fe_3_O_4_@C@CdTe QDs	LBL assembly	Water	ND	1–10 μM	Highly adsorptive; simple removal and detection; recyclable; non-specificity	[120]
Hg^2+^	Fe_3_O_4_@CQDs	One-pot method	Lake/tap water and drinks	0.3 nM	0.003–0.01 μM	Multifunctional separation, enrichment, detection and removal; ultra-sensitive; good recoveries and reproducibility	[127]
Hg^2+^	Fe_3_O_4_@SiO_2_@GQDs	LBL assembly	Tap/well/river water	30 nM	0.1–70 μM	Favorable sensitivity and selectivity; strong affinity and fast response; recyclable	[129]
Hg^2+^	Fe_3_O_4_@SiO_2_@CdTe QDs-Rh6G	Template embedding	Deionized/tap water	2.5 nM	7–900 nM	Highly selective, sensitive, and regenerative ratiometric fluorescent sensing	[130]
Cu^2+^, Fe^3+^	CuFeS_2_ QDs	One-pot method	Water	1.98, 2.15 μM	0–30 μM; 0–45 μM	High throughput detection; non-specificity	[134]
Co^2+^, Ni^2+^, Pb^2+^	CaCO_3_-Fe_3_O_4_-AgInS_2_/ZnS QDs	LBL assembly	Water	10, 100, 100 nM	ND	Quick fluorescence response; high-throughput detection; non-specificity	[126]

ND: no data available.

**Table 2:** Detection performances of MNPs@QDs-based sensors for foodborne pathogens.

Analytes	Nanocomposites	Synthetic Strategy	Samples	LOD (CFU/mL)	Linear Range (CFU/mL)	Remarks	Reference
*E. coli*	Fe_3_O_4_@SiO_2_@QDs	LBL assembly	Milk	2.39 × 10^2^	2.5 × 10^2^–5 × 10^5^	Simple and rapid; increased sensitivity; good anti-interference property	[139]
*S. typhi*	Fe_3_O_4_@PEI@CdSe/ZnS QDs	LBL assembly	Water, milk	3.75 × 10^3^	1.88 × 10^4^–1.88 × 10^7^	Multifunctional target separation and enrichment; multi-signal readout; double formats of quantitation; good anti-interference property	[140]
*S. typhi*	Fe_3_O_4_@CS@CQDs	LBL assembly	Lettuce	1.38 × 10^2^	10^3^–10^6^	Favorable sensitivity and selectivity; rapid and simple; inexpensive and eco-friendly	[144]
*S. typhi*	Fe_3_O_4_@SiO_2_@CdTe/ZnS QDs	LBL assembly	Milk	1.7 × 10^2^	ND	Excellent sensitivity, selectivity, stability, and reproducibility; more time-consumption	[148]
*E. coli*, *S. typhi*	Fe2O3@SiO2@CdSe/ZnS QDs	LBL assembly	Milk	16; 25	40–10^8^; 63–10^8^	Magnetic encoded for high throughput detection; excellent sensitivity and stability; controllable	[145]
*S. agalactiae*	Fe_3_O_4_@SiO_2_@CdTe QDs	LBL assembly	Milk	10^2^	ND	Good sensitivity and selectivity; distinguished by naked-eye; complicated operations and insufficient	[141]
*Alicyclobacillus* spp.	Fe_3_O_4_@SiO_2_@CdSe/ZnS QDs	LBL assembly	Apple juice	10^4^	10^4^–10^7^	Good selectivity; more time-consuming	[147]

ND: no data available.

**Table 3:** Detection performances of MNPs@QDs-based sensors for toxins.

Analytes	Nanocomposites	Synthetic Strategy	Samples	LOD	Linear Range	Remarks	Reference
AFB_1_	PMMA-PMAO (OA-MNPs@OC-QDs)	Microemulsion	Dark soy sauce	3 pg/mL	5–150 pg/mL	Enhanced sensitivity and accuracy; rapid and low cost; good anti-interference ability	[99]
BoNT/A, SEB	Fe_3_O_4_@PEI@CdSe/Zn QDs	LBL assembly	Milk, grape juice	BoNT/A:2.52 pg/mL SEB:2.86 pg/mL	Both 1–100 pg/mL	Sensitive and high-throughput; favorable selectivity and reproducibility; time-saving	[152]

**Table 4:** Detection performances of MNPs@QDs-based sensors for pesticides, antibiotics, and illegal additives.

Analytes	Nanocomposites	Synthetic Strategy	Samples	LOD	Linear Range	Remarks	Reference
**Pesticides**							
Trichlorfon	Fe_3_O_4_@SiO_2_@CdTe QDs-MIPs	LBL assembly	Rape	30 ng/g	ND	High adsorption capacity; good selectivity and reproducibility; low sensitivity	[154]
4-nitrophenol	Fe_3_O_4_@SiO_2_@CQDs	Microemulsion	Water/Fish	23.45 nM	0.08–10 μM	High selectivity and sensitivity; excellent stability and reusability; good anti-interference	[155]
N-Nitrosodiphenylamine	Fe_3_O_4_@SiO_2_@Mn-ZnS QDs-MIPs	Microemulsion	Tap water/Seawater	0.69 μM	0–120 μM	High selectivity; complicated operations	[156]
**Antibiotics**							
	Fe_3_O_4_@SiO_2_@Mn-ZnS QDs-MIPs	LBL assembly	Fish/Milk	0.8 ng/mL	1–90 ng/mL	Easy operation; quick response; rapid detection and cost-effective	[160]
Cefoperazone	PGr/CdTe QDs/Fe_3_O_4_@SiO_2_/MIPs	LBL assembly	Milk	0.09 ng/mL	0.1–25 ng/mL	Ultra-sensitive, selective, and rapid; cost-effective and user-friendly	[163]
Mafenide, sulfisoxazole	MIL101-MMIP-GQDs; MIL101-MMIP-CdTe QDs	LBL assembly	Milk	Both 0.1 ng/mL	Both 0.1–25 ng/mL	Excellent selectivity and sensitivity; high-throughput; time-saving	[164]
**Illegal additives**							
Clenbuterol	Fe_3_O_4_@SiO_2_@CdSe/ZnS QDs	LBL assembly	Swine urine	0.22 ng/mL	0.25–5 ng/mL	High sensitivity, accuracy, and specificity; good matrix tolerance; rapid and portable	[166]
Malachite green	CdTe QDs/nano-Fe_3_O_4_@MIPs	Microemulsion	Fish	0.014 μM	0.025–1.5 μM	Good sensitivity and reproducibility; non-specificity	[167]
Bisphenol A	Fe_3_O_4_@SiO_2_@CdSe QDs	Template embedding	Water	0.34 nM	10^−9^–10^−4^M	High sensitivity and stability; more time-consumption	[168]

ND: no data available.

## Data Availability

Not applicable.

## References

[1] Bhavadharini B., Kavimughil M., Malini B., Vallath A., Prajapati H.K., Sunil C.K. (2022). Recent Advances in Biosensors for Detection of Chemical Contaminants in Food—A Review. Food Anal. Methods.

[2] Romero-Gonzalez R. (2015). Food safety: How analytical chemists ensure it. Anal. Methods.

[3] Pena-Rosas J.P., De-Regil L.M., Rogers L.M., Bopardikar A., Panisset U. (2012). Translating Research into Action: WHO Evidence-Informed Guidelines for Safe and Effective Micronutrient Interventions. J. Nutr..

[4] Barzegar F., Kamankesh M., Mohammadi A. (2021). Recent Development in Formation, Toxic Effects, Human Health and Analytical Techniques of Food Contaminants. Food Rev. Int..

[5] Mejia-Carmona K., Maciel E.V.S., Lancas F.M. (2020). Miniaturized liquid chromatography applied to the analysis of residues and contaminants in food: A review. Electrophoresis.

[6] Xu M.-L., Gao Y., Wang X., Han X.X., Zhao B. (2021). Comprehensive Strategy for Sample Preparation for the Analysis of Food Contaminants and Residues by GC-MS/MS: A Review of Recent Research Trends. Foods.

[7] Masia A., Morales Suarez-Varela M., Llopis-Gonzalez A., Pico Y. (2016). Determination of pesticides and veterinary drug residues in food by liquid chromatography-mass spectrometry: A review. Anal. Chim. Acta.

[8] Lv M., Liu Y., Geng J., Kou X., Xin Z., Yang D. (2018). Engineering nanomaterials-based biosensors for food safety detection. Biosens. Bioelectron..

[9] Bhardwaj N., Bhardwaj S.K., Nayak M.K., Mehta J., Kim K.-H., Deep A. (2017). Fluorescent nanobiosensors for the targeted detection of foodborne bacteria. Trends Anal. Chem..

[10] Bobrinetskiy I.I., Knezevic N.Z. (2018). Graphene-based biosensors for on-site detection of contaminants in food. Anal. Methods.

[11] Sharma R., Ragavan K.V., Thakur M.S., Raghavarao K.S.M.S. (2015). Recent advances in nanoparticle based aptasensors for food contaminants. Biosens. Bioelectron..

[12] Marrubini G., Appelblad P., Maietta M., Papetti A. (2018). Hydrophilic interaction chromatography in food matrices analysis: An updated review. Food Chem..

[13] Barreiro J.C., Luiz A.L., Fernandes Maciel S.C., Soares Maciel E.V., Lancas F.M. (2015). Recent approaches for on-line analysis of residues and contaminants in food matrices: A review. J. Sep. Sci..

[14] Khan R., Rehman A., Hayat A., Andreescu S. (2019). Magnetic Particles-Based Analytical Platforms for Food Safety Monitoring. Magnetochemistry.

[15] Gao Q., Luo D., Bai M., Chen Z.-W., Feng Y.-Q. (2011). Rapid Determination of Estrogens in Milk Samples Based on Magnetite Nanoparticles/Polypyrrole Magnetic Solid-Phase Extraction Coupled with Liquid Chromatography-Tandem Mass Spectrometry. J. Agric. Food Chem..

[16] Yu X., Zhong T., Zhang Y., Zhao X., Xiao Y., Wang L., Liu X., Zhang X. (2021). Design, Preparation, and Application of Magnetic Nanoparticles for Food Safety Analysis: A Review of Recent Advances. J. Agric. Food Chem..

[17] Mahmoudi M., Sant S., Wang B., Laurent S., Sen T. (2011). Superparamagnetic iron oxide nanoparticles (SPIONs): Development, surface modification and applications in chemotherapy. Adv. Drug Deliv. Rev..

[18] Tan J., Wang T., Li Y., Xu S., Chen S., Hao H. (2021). Review on functionalized magnetic nanoparticles for the pretreatment of organophosphorus pesticides. Green Process. Synth..

[19] Peltomaa R., Barderas R., Benito-Pena E., Moreno-Bondi M.C. (2022). Recombinant antibodies and their use for food immunoanalysis. Anal. Bioanal. Chem..

[20] Yan M., Li H., Li M., Cao X., She Y., Chen Z. (2021). Advances in Surface-Enhanced Raman Scattering-Based Aptasensors for Food Safety Detection. J. Agric. Food Chem..

[21] Villa C.C., Sanchez L.T., Valencia G.A., Ahmed S., Gutierrez T.J. (2021). Molecularly imprinted polymers for food applications: A review. Trends Food Sci. Technol..

[22] Chen L., Cheng Z., Luo M., Wang T., Zhang L., Wei J., Wang Y., Li P. (2021). Fluorescent noble metal nanoclusters for contaminants analysis in food matrix. Crit. Rev. Food Sci. Nutr..

[23] He H., Sun D., Wu Z., Pu H., Wei Q. (2022). On-off-on fluorescent nanosensing: Materials, detection strategies and recent food applications. Trends Food Sci. Technol..

[24] Li T., Li Z., Huang T., Tian L. (2021). Carbon quantum dot-based sensors for food safety. Sens. Actuators A.

[25] Wu Y., Sun J., Huang X., Lai W., Xiong Y. (2021). Ensuring food safety using fluorescent nanoparticles-based immunochromatographic test strips. Trends Food Sci. Technol..

[26] Ledentsov N.N., Ustinov V.M., Shchukin V.A., Kop’ev P.S., Alferov Z.I., Bimberg D. (1998). Quantum dot heterostructures: Fabrication, properties, lasers (Review). Semiconductors.

[27] Galstyan V. (2021). “Quantum dots: Perspectives in next-generation chemical gas sensors”—A review. Anal. Chim. Acta.

[28] Kaur M., Kaur M., Sharma V.K. (2018). Nitrogen-doped graphene and graphene quantum dots: A review onsynthesis and applications in energy, sensors and environment. Adv. Colloid Interface Sci..

[29] Khan Z.G., Patil P.O. (2020). A comprehensive review on carbon dots and graphene quantum dots based fluorescent sensor for biothiols. Microchem. J..

[30] Molaei M.J. (2020). Principles, mechanisms, and application of carbon quantum dots in sensors: A review. Anal. Methods.

[31] Wang X., Cheng L. (2019). Multifunctional two-dimensional nanocomposites for photothermal-based combined cancer therapy. Nanoscale.

[32] Kaewsaneha C., Tangboriboonrat P., Polpanich D., Elaissari A. (2015). Multifunctional Fluorescent-Magnetic Polymeric Colloidal Particles: Preparations and Bioanalytical Applications. ACS Appl. Mater. Interfaces.

[33] Koole R., Mulder W.J.M., van Schooneveld M.M., Strijkers G.J., Meijerink A., Nicolay K. (2009). Magnetic quantum dots for multimodal imaging. Wiley Interdiscip. Rev. Nanomed. Nanobiotechnol..

[34] Mahajan K.D., Fan Q., Dorcena J., Ruan G., Winter J.O. (2013). Magnetic quantum dots in biotechnology–synthesis and applications. Biotechnol. J..

[35] Roullier V., Marchi-Artzner V., Cador O., Dorson F., Aubert T., Cordier S., Molard Y., Grasset F., Mornet S., Haneda H. (2010). Synthesis and characterisation of magnetic-luminescent composite colloidal nanostructures. Int. J. Nanotechnol..

[36] Xia H., Tong R., Song Y., Xiong F., Li J., Wang S., Fu H., Wen J., Li D., Zeng Y. (2017). Synthesis and bio-applications of targeted magnetic-fluorescent composite nanoparticles. J. Nanopart. Res..

[37] Acharya A. (2013). Luminescent Magnetic Quantum Dots for In Vitro/In Vivo Imaging and Applications in Therapeutics. J. Nanosci. Nanotechnol..

[38] Jing L., Ding K., Kershaw S.V., Kempson I.M., Rogach A.L., Gao M. (2014). Magnetically Engineered Semiconductor Quantum Dots as Multimodal Imaging Probes. Adv. Mater..

[39] Tufani A., Qureshi A., Niazi J.H. (2021). Iron oxide nanoparticles based magnetic luminescent quantum dots (MQDs) synthesis and biomedical/biological applications: A review. Mater. Sci. Eng. C.

[40] Kim H., Achermann M., Balet L.P., Hollingsworth J.A., Klimov V.I. (2005). Synthesis and characterization of Co/CdSe core/shell nanocomposites: Bifunctional magnetic-optical nanocrystals. J. Am. Chem. Soc..

[41] Zhou S., Chen Q., Hu X., Zhao T. (2012). Bifunctional luminescent superparamagnetic nanocomposites of CdSe/CdS-Fe_3_O_4_ synthesized via a facile method. J. Mater. Chem..

[42] Bhandari S., Khandelia R., Pan U.N., Chattopadhyay A. (2015). Surface Complexation-Based Biocompatible Magnetofluorescent Nanoprobe for Targeted Cellular Imaging. ACS Appl. Mater. Interfaces.

[43] Wu Y., Zou H., Zhang Y., Mou M., Niu Q., Yan Z., Liao S. (2020). The Loading of Luminescent Magnetic Nanocomposites Fe_3_O_4_@Polyaniline/Carbon Dots for Methotrexate and Its Release Behavior In Vitro. J. Nanosci. Nanotechnol..

[44] Lee J.-S., Bodnarchuk M.I., Shevchenko E.V., Talapin D.V. (2010). “Magnet-in-the-Semiconductor” FePt-PbS and FePt-PbSe Nanostructures: Magnetic Properties, Charge Transport, and Magnetoresistance. J. Am. Chem. Soc..

[45] Gu H.W., Zheng R.K., Zhang X.X., Xu B. (2004). Facile one-pot synthesis of bifunctional heterodimers of nanoparticles: A conjugate of quantum dot and magnetic nanoparticles. J. Am. Chem. Soc..

[46] Selvan S.T., Patra P.K., Ang C.Y., Ying J.Y. (2007). Synthesis of silica-coated semiconductor and magnetic quantum dots and their use in the imaging of live cells. Angew. Chem. Int. Ed..

[47] Lin A.W.H., Ang C.Y., Patra P.K., Han Y., Gu H., Le Breton J.-M., Juraszek J., Chiron H., Papaefthymiou G.C., Selvan S.T. (2011). Seed-mediated synthesis, properties and application of gamma-Fe_2_O_3_-CdSe magnetic quantum dots. J. Solid State Chem..

[48] McDaniel H., Shim M. (2009). Size and Growth Rate Dependent Structural Diversification of Fe_3_O_4_/CdS Anisotropic Nanocrystal Heterostructures. ACS Nano.

[49] Gao J., Zhang W., Huang P., Zhang B., Zhang X., Xu B. (2008). Intracellular spatial control of fluorescent magnetic nanoparticles. J. Am. Chem. Soc..

[50] Du G.H., Liu Z.L., Lu Q.H., Xia X., Jia L.H., Yao K.L., Chu Q., Zhang S.M. (2006). Fe_3_O_4_/CdSe/ZnS magnetic fluorescent bifunctional nanocomposites. Nanotechnology.

[51] Hines M.A., Guyot-Sionnest P. (1996). Synthesis and characterization of strongly luminescing ZnS-Capped CdSe nanocrystals. J. Phys. Chem..

[52] Deng S., Ruan G., Han N., Winter J.O. (2010). Interactions in fluorescent-magnetic heterodimer nanocomposites. Nanotechnology.

[53] Beaune G., Dubertret B., Clement O., Vayssettes C., Cabuil V., Menager C. (2007). Giant vesicles containing magnetic nanoparticles and quantum dots: Feasibility and tracking by fiber confocal fluorescence microscopy. Angew. Chem. Int. Ed..

[54] Ruan G., Vieira G., Henighan T., Chen A., Thakur D., Sooryakumar R., Winter J.O. (2010). Simultaneous Magnetic Manipulation and Fluorescent Tracking of Multiple Individual Hybrid Nanostructures. Nano Lett..

[55] Park J.-H., von Maltzahn G., Ruoslahti E., Bhatia S.N., Sailor M.J. (2008). Micellar hybrid nanoparticles for simultaneous magnetofluorescent imaging and drug delivery. Angew. Chem. Int. Ed..

[56] Kim J., Lee J.E., Lee J., Yu J.H., Kim B.C., An K., Hwang Y., Shin C.H., Park J.G., Kim J. (2006). Magnetic fluorescent delivery vehicle using uniform mesoporous silica spheres embedded with monodisperse magnetic and semiconductor nanocrystals. J. Am. Chem. Soc..

[57] Sun L., Zang Y., Sun M.D., Wang H.G., Zhu X.J., Xu S.F., Yang Q.B., Li Y.X., Shan Y.M. (2010). Synthesis of magnetic and fluorescent multifunctional hollow silica nanocomposites for live cell imaging. J. Colloid Interface Sci..

[58] Xiao Q., Xiao C. (2009). Preparation and Characterization of Silica-Coated Magnetic-Fluorescent Bifunctional Microspheres. Nanoscale Res. Lett..

[59] Yi D.K., Selvan S.T., Lee S.S., Papaefthymiou G.C., Kundaliya D., Ying J.Y. (2005). Silica-coated nanocomposites of magnetic nanoparticles and quantum dots. J. Am. Chem. Soc..

[60] Ruan J., Wang K., Song H., Xu X., Ji J.J., Cui D.X. (2011). Biocompatibility of hydrophilic silica-coated CdTe quantum dots and magnetic nanoparticles. Nanoscale Res. Lett..

[61] Kyeong S., Jeong C., Kim H.Y., Hwang D.W., Kang H., Yang J.-K., Lee D.S., Jun B.-H., Lee Y.-S. (2015). Fabrication of mono-dispersed silica-coated quantum dot-assembled magnetic nanoparticles. RSC Adv..

[62] Huang L., Zhang Y., Liao T., Xu K., Jiang C., Zhuo D., Wang Y., Wen H.-M., Wang J., Ao L. (2021). Compact Magneto-Fluorescent Colloids by Hierarchical Assembly of Dual-Components in Radial Channels for Sensitive Point-of-Care Immunoassay. Small.

[63] Tan Y.F., Chandrasekharan P., Maity D., Yong C.X., Chuang K.-H., Zhao Y., Wang S., Ding J., Feng S.-S. (2011). Multimodal tumor imaging by iron oxides and quantum dots formulated in poly (lactic acid)-D-alpha-tocopheryl polyethylene glycol 1000 succinate nanoparticles. Biomaterials.

[64] Xie H.Y., Zuo C., Liu Y., Zhang Z.L., Pang D.W., Li X.L., Gong J.P., Dickinson C., Zhou W.Z. (2005). Cell-targeting multifunctional nanospheres with both fluorescence and magnetism. Small.

[65] Li Y.-H., Song T., Liu J.-Q., Zhu S.-J., Chang J. (2011). An efficient method for preparing high-performance multifunctional polymer beads simultaneously incorporated with magnetic nanoparticles and quantum dots. J. Mater. Chem..

[66] Wilson R., Spiller D.G., Prior I.A., Veltkamp K.J., Hutchinson A. (2007). A Simple Method for Preparing Spectrally Encoded Magnetic Beads for Multiplexed Detection. ACS Nano.

[67] Wang C., Wang L., Yang W. (2009). Preparation and characterization of functional inorganic/organic composite microspheres via electrostatic interaction. J. Colloid Interface Sci..

[68] Li L., Choo E.S.G., Liu Z., Ding J., Xue J. (2008). Double-layer silica core-shell nanospheres with superparamagnetic and fluorescent functionalities. Chem. Phys. Lett..

[69] Yin N., Wang X., Yang T., Ding Y., Li L., Zhao S., Li P., Xu X., Zhu L. (2021). Multifunctional Fe_3_O_4_ cluster@ quantum dot-embedded mesoporous SiO2 nanoplatform probe for cancer cell fluorescence-labelling detection and photothermal therapy. Ceram. Int..

[70] Yoo J.H., Kim J.S. (2013). The Preparation of Core-Shell Magnetic Silica Nanospheres for Enhancing Magnetism and Fluorescence Intensity. J. Nanosci. Nanotechnol..

[71] Sathe T.R., Agrawal A., Nie S. (2006). Mesoporous silica beads embedded with semiconductor quantum dots and iron oxide nanocrystals: Dual-function microcarriers for optical encoding and magnetic separation. Anal. Chem..

[72] Xie H.-Y., Xie M., Zhang Z.-L., Long Y.-M., Liu X., Tang M.-L., Pang D.-W., Tan Z., Dickinson C., Zhou W. (2007). Wheat germ agglutinin-modified trifunctional nanospheres for cell recognition. Bioconjugate Chem..

[73] Zebli B., Susha A.S., Sukhorukov G.B., Rogach A.L., Parak W.J. (2005). Magnetic Targeting and Cellular Uptake of Polymer Microcapsules Simultaneously Functionalized with Magnetic and Luminescent Nanocrystals. Langmuir.

[74] Li Z., Wang G., Shen Y., Guo N., Ma N. (2018). DNA-Templated Magnetic Nanoparticle-Quantum Dot Polymers for Ultrasensitive Capture and Detection of Circulating Tumor Cells. Adv. Funct. Mater..

[75] Zou W.-S., Yang J., Yang T.-T., Hu X., Lian H.-Z. (2012). Magnetic-room temperature phosphorescent multifunctional nanocomposites as chemosensor for detection and photo-driven enzyme mimetics for degradation of 2,4,6-trinitrotoluene. J. Mater. Chem..

[76] Ortgies D.H., de la Cueva L., del Rosal B., Sanz-Rodríguez F., Fernández N., Iglesias-de la Cruz M.C., Salas G., Cabrera D., Teran F.J., Jaque D. (2016). In Vivo Deep Tissue Fluorescence and Magnetic Imaging Employing Hybrid Nanostructures. ACS Appl. Mater. Interfaces.

[77] Xiao L.-H., Wang T., Zhao T.-Y., Zheng X., Sun L.-Y., Li P., Liu F.-Q., Gao G., Dong A. (2013). Fabrication of Magnetic-Antimicrobial-Fluorescent Multifunctional Hybrid Microspheres and Their Properties. Int. J. Mol. Sci..

[78] Fan H.-M., Olivo M., Shuter B., Yi J.-B., Bhuvaneswari R., Tan H.-R., Xing G.-C., Ng C.-T., Liu L., Lucky S.S. (2010). Quantum Dot Capped Magnetite Nanorings as High Performance Nanoprobe for Multiphoton Fluorescence and Magnetic Resonance Imaging. J. Am. Chem. Soc..

[79] Hong X., Li J., Wang M.J., Xu J.J., Guo W., Li J.H., Bai Y.B., Li T.J. (2004). Fabrication of magnetic luminescent nanocomposites by a layer-by-layer self-assembly approach. Chem. Mater..

[80] Wang C.W., Shen W.Z., Rong Z., Liu X.X., Gu B., Xiao R., Wang S.Q. (2020). Layer-by-layer assembly of magnetic-core dual quantum dot-shell nanocomposites for fluorescence lateral flow detection of bacteria. Nanoscale.

[81] Chen B., Zhang H., Zhai C., Du N., Sun C., Xue J., Yang D., Huang H., Zhang B., Xie Q. (2010). Carbon nanotube-based magnetic-fluorescent nanohybrids as highly efficient contrast agents for multimodal cellular imaging. J. Mater. Chem..

[82] Sun X., Ding K., Hou Y., Gao Z., Yang W., Jing L., Gao M. (2013). Bifunctional Superparticles Achieved by Assembling Fluorescent CuInS_2_@ZnS Quantum Dots and Amphibious Fe_3_O_4_ Nanocrystals. J. Phys. Chem. C.

[83] Wang D., He J., Rosenzweig N., Rosenzweig Z. (2004). Superparamagnetic Fe_2_O_3_ Beads−CdSe/ZnS Quantum Dots Core−Shell Nanocomposite Particles for Cell Separation. Nano Lett..

[84] Nikitin M.P., Zdobnova T.A., Lukash S.V., Stremovskiy O.A., Deyev S.M. (2010). Protein-assisted self-assembly of multifunctional nanoparticles. Proc. Natl. Acad. Sci. USA.

[85] Shibu E.S., Ono K., Sugino S., Nishioka A., Yasuda A., Shigeri Y., Wakida S.-i., Sawada M., Biju V. (2013). Photouncaging Nanoparticles for MRI and Fluorescence Imaging in Vitro and in Vivo. ACS Nano.

[86] Song E., Han W., Xu H., Jiang Y., Cheng D., Song Y., Swihart M.T. (2014). Magnetically Encoded Luminescent Composite Nanoparticles through Layer-by-Layer Self-Assembly. Chem.—Eur. J..

[87] Rong Z., Bai Z.K., Li J.N., Tang H., Shen T.Y., Wang Q., Wang C.W., Xiao R., Wang S.Q. (2019). Dual-color magnetic-quantum dot nanobeads as versatile fluorescent probes in test strip for simultaneous point-of-care detection of free and complexed prostate-specific antigen. Biosens. Bioelectron..

[88] Wang C., Cheng X., Liu L., Zhang X., Yang X., Zheng S., Rong Z., Wang S. (2021). Ultrasensitive and Simultaneous Detection of Two Specific SARS-CoV-2 Antigens in Human Specimens Using Direct/Enrichment Dual-Mode Fluorescence Lateral Flow Immunoassay. ACS Appl. Mater. Interfaces.

[89] Dong S., Wang S., Wang X., Zhai L. (2020). Superparamagnetic nanocomposite Fe_3_O_4_@SiO_2_-NH_2_/CQDs as fluorescent probe for copper (II) detection. Mater. Lett..

[90] Zhang X., Tang B., Li Y., Liu C., Jiao P., Wei Y. (2021). Molecularly Imprinted Magnetic Fluorescent Nanocomposite-Based Sensor for Selective Detection of Lysozyme. Nanomaterials.

[91] Li C.-L., Huang B.-R., Chang J.-Y., Chen J.-K. (2015). Bifunctional superparamagnetic-luminescent core-shell-satellite structured microspheres: Preparation, characterization, and magnetodisplay application. J. Mater. Chem. C.

[92] Koc K., Karakus B., Rajar K., Alveroglu E. (2017). Synthesis and characterization. of ZnS@Fe_3_O_4_ fluorescent-magnetic bifunctional nanospheres. Superlattices Microstruct..

[93] Wang K., Xu X., Li Y., Rong M., Wang L., Lu L., Wang J., Zhao F., Sun B., Jiang Y. (2021). Preparation Fe_3_O_4_@chitosan-graphene quantum dots nanocomposites for fluorescence and magnetic resonance imaging. Chem. Phys. Lett..

[94] Wang Z., Jiang X., Liu W., Lu G., Huang X. (2019). A rapid and operator-safe powder approach for latent fingerprint detection using hydrophilic Fe_3_O_4_@SiO_2_-CdTe nanoparticles. Sci. China Chem..

[95] Wang M., Fei X.F., Lv S.W., Sheng Y., Zou H.F., Song Y.H., Yan F., Zhu Q.L., Zheng K.Y. (2018). Synthesis and characterization of a flexible fluorescent magnetic Fe_3_O_4_@SiO_2_/CdTe-NH_2_ nanoprobe. J. Inorg. Biochem..

[96] Zhang Y., Wang S.-N., Ma S., Guan J.-J., Li D., Zhang X.-D., Zhang Z.-D. (2008). Self-assembly multifunctional nanocomposites with Fe_3_O_4_ magnetic core and CdSe/ZnS quantum dots shell. J. Biomed. Mater. Res. Part A.

[97] Chen O., Riedemann L., Etoc F., Herrmann H., Coppey M., Barch M., Farrar C.T., Zhao J., Bruns O.T., Wei H. (2014). Magneto-fluorescent core-shell supernanoparticles. Nat. Commun..

[98] Piao Y., Burns A., Kim J., Wiesner U., Hyeon T. (2008). Designed Fabrication of Silica-Based Nanostructured Particle Systems for Nanomedicine Applications. Adv. Funct. Mater..

[99] Guo L., Shao Y., Duan H., Ma W., Leng Y., Huang X., Xiong Y. (2019). Magnetic Quantum Dot Nanobead-Based Fluorescent Immunochromatographic Assay for the Highly Sensitive Detection of Aflatoxin B_1_ in Dark Soy Sauce. Anal. Chem..

[100] Guo S., Chen Y.-Q., Lu N.-N., Wang X.-Y., Xie M., Sui W.-P. (2014). Ultrasonication-assisted one-step self-assembly preparation of biocompatible fluorescent-magnetic nanobeads for rare cancer cell detection. Nanotechnology.

[101] Kim J., Lee J.E., Lee S.H., Yu J.H., Lee J.H., Park T.G., Hyeon T. (2008). Designed fabrication of a multifunctional polymer nanomedical platform for simultaneous cancer-targeted imaging and magnetically guided drug delivery. Adv. Mater..

[102] Leng Y., Wu W., Li L., Lin K., Sun K., Chen X., Li W. (2016). Magnetic/Fluorescent Barcodes Based on Cadmium-Free Near-Infrared-Emitting Quantum Dots for Multiplexed Detection. Adv. Funct. Mater..

[103] Lan J., Chen J., Li N., Ji X., Yu M., He Z. (2016). Microfluidic generation of magnetic-fluorescent Janus microparticles for biomolecular detection. Talanta.

[104] Zhou C., Wang Z., Xia J., Via B.K., Zhang F., Xia Y., Li Y. (2012). A simplistic one-pot method to produce magnetic graphene-CdS nanocomposites. Comptes Rendus Chim..

[105] Maleki S., Madrakian T., Afkhami A. (2019). Magnetic solid-phase extraction of codeine in a biological sample utilizing Fe_3_O_4_/CDs/Lys nanocomposite as an efficient adsorbent. J. Iran. Chem. Soc..

[106] Liu X., Jiang H., Ye J., Zhao C., Gao S., Wu C., Li C., Li J., Wang X. (2016). Nitrogen-Doped Carbon Quantum Dot Stabilized Magnetic Iron Oxide Nanoprobe for Fluorescence, Magnetic Resonance, and Computed Tomography Triple-Modal In Vivo Bioimaging. Adv. Funct. Mater..

[107] Li B., Wang X., Guo Y., Iqbal A., Dong Y., Li W., Liu W., Qin W., Chen S., Zhou X. (2016). One-pot synthesis of polyamines improved magnetism and fluorescence Fe_3_O_4_-carbon dots hybrid NPs for dual modal imaging. Dalton Trans..

[108] Irmania N., Dehvari K., Gedda G., Tseng P.-J., Chang J.-Y. (2020). Manganese-doped green tea-derived carbon quantum dots as a targeted dual imaging and photodynamic therapy platform. J. Biomed. Mater. Res. Part B.

[109] Ji Z., Ai P., Shao C., Wang T., Yan C., Ye L., Gu W. (2018). Manganese-Doped Carbon Dots for Magnetic Resonance/Optical Dual-Modal Imaging of Tiny Brain Glioma. ACS Biomater. Sci. Eng..

[110] Yao Y.-Y., Gedda G., Girma W.M., Yen C.-L., Ling Y.-C., Chang J.-Y. (2017). Magnetofluorescent Carbon Dots Derived from Crab Shell for Targeted Dual-Modality Bioimaging and Drug Delivery. ACS Appl. Mater. Interfaces.

[111] Liao H., Wang Z., Chen S., Wu H., Ma X., Tan M. (2015). One-pot synthesis of gadolinium(III) doped carbon dots for fluorescence/magnetic resonance bimodal imaging. RSC Adv..

[112] Yu C., Xuan T., Chen Y., Zhao Z., Liu X., Lian G., Li H. (2016). Gadolinium-doped carbon dots with high quantum yield as an effective fluorescence and magnetic resonance bimodal imaging probe. J. Alloys Compd..

[113] Wu F., Yue L., Yang L., Wang K., Liu G., Luo X., Zhu X. (2019). Ln(III) chelates-functionalized carbon quantum dots: Synthesis, optical studies and multimodal bioimaging applications. Colloids Surf. B.

[114] Moro L., Turemis M., Marini B., Ippodrino R., Giardi M.T. (2017). Better together: Strategies based on magnetic particles and quantum dots for improved biosensing. Biotechnol. Adv..

[115] Li Y., Chen Y., Yu H., Tian L., Wang Z. (2018). Portable and smart devices for monitoring heavy metal ions integrated with nanomaterials. TrAC Trends Anal. Chem..

[116] Liu L., Xiao L., Zhu H., Shi X. (2012). Preparation of magnetic and fluorescent bifunctional chitosan nanoparticles for optical determination of copper ion. Microchim. Acta.

[117] Xie C., Xiao L., Peng S., Shi X. (2014). Preparation of novel magnetic and fluorescent CS-Fe_3_O_4_@CdSeS nanoparticles for simultaneous removal and optical determination of trace copper ions. New J. Chem..

[118] Kumar A., Chowdhuri A.R., Laha D., Chandra S., Karmakar P., Sahu S.K. (2016). One-pot synthesis of carbon dot-entrenched chitosan-modified magnetic nanoparticles for fluorescence-based Cu^2+^ ion sensing and cell imaging. RSC Adv..

[119] Rafiee F., Tajfar N., Mohammadnejad M. (2021). The synthesis and efficiency investigation of a boronic acid-modified magnetic chitosan quantum dot nanocomposite in the detection of Cu^2+^ ions. Int. J. Biol. Macromol..

[120] Wang H., Sun L., Li Y., Fei X., Sun M., Zhang C., Li Y., Yang Q. (2011). Layer-by-Layer Assembled Fe_3_O_4_@C@CdTe Core/Shell Microspheres as Separable Luminescent Probe for Sensitive Sensing of Cu^2+^ Ions. Langmuir.

[121] Li L., Jia C., Wang F.J., Fan H.L., Jiao W.Z., Shao Z.Q. (2018). Facile synthesis of magnetic fluorescent nanoparticles: Adsorption and selective detection of Hg(II) in water. J. Mater. Chem. C.

[122] Yang C.H., Ding Y.L., Qian J. (2018). Design of magnetic-fluorescent based nanosensor for highly sensitive determination and removal of HG_2+_. Ceram. Int..

[123] Zhai X., Gong Y., Yang W., Kang H., Zhang X. (2015). Mn-doped CdS/ZnS/CdS QD-based fluorescent nanosensor for rapid, selective, and ultrasensitive detection of copper(ii) ion. RSC Adv..

[124] Yang P., Zhu B., Zhao J., Yu H., Yan L., Wei Q., Du B. (2013). A novel reusable glutathione-modified magnetic fluorescent nanosensor for highly sensitive determination and removal of Cu^2+^. Inorg. Chim. Acta.

[125] Zhu B., Yang P., Yu H., Yan L., Wei Q., Du B. (2013). Development of a novel water-soluble magnetic fluorescent nanoparticle for the selective detection and removal of Cu^2+^. Nanotechnology.

[126] Kurshanov D.A., Khavlyuk P.D., Baranov M.A., Dubavik A., Rybin A.V., Fedorov A.V., Baranov A.V. (2020). Magneto-Fluorescent Hybrid Sensor CaCO_3_-Fe_3_O_4_-AgInS_2_/ZnS for the Detection of Heavy Metal Ions in Aqueous Media. Materials.

[127] Xie R., Qu Y., Tang M., Zhao J., Chua S., Li T., Zhang F., Wheatley A.E.H., Chai F. (2021). Carbon dots-magnetic nanocomposites for the detection and removal of Hg^2+^. Food Chem..

[128] Xu J., Wang Y., Hu S. (2017). Nanocomposites of graphene and graphene oxides: Synthesis, molecular functionalization and application in electrochemical sensors and biosensors. A review. Microchim. Acta.

[129] Alvand M., Shemirani F. (2017). A Fe_3_O_4_@SiO_2_@graphene quantum dot core-shell structured nanomaterial as a fluorescent probe and for magnetic removal of mercury(II) ion. Microchim. Acta.

[130] Wang Y., Tang M., Shen H., Che G., Qiao Y., Liu B., Wang L. (2018). Recyclable Multifunctional Magnetic Mesoporous Silica Nanocomposite for Ratiometric Detection, Rapid Adsorption, and Efficient Removal of Hg(II). ACS Sustain. Chem. Eng..

[131] Ahmadian-Fard-Fini S., Ghanbari D., Salavati-Niasari M. (2019). Photoluminescence carbon dot as a sensor for detecting of Pseudomonas aeruginosa bacteria: Hydrothermal synthesis of magnetic hollow NiFe_2_O_4_-carbon dots nanocomposite material. Compos. Part B.

[132] Ahmadian-Fard-Fini S., Ghanbari D., Amiri O., Salavati-Niasari M. (2021). Green sonochemistry assisted synthesis of hollow magnetic and photoluminescent MgFe_2_O_4_-carbon dot nanocomposite as a sensor for toxic Ni(ii), Cd(ii) and Hg(ii) ions and bacteria. RSC Adv..

[133] Yue L., Li H., Liu Q., Guo D., Chen J., Sun Q., Xu Y., Wu F. (2019). Manganese-doped carbon quantum dots for fluorometric and magnetic resonance (dual mode) bioimaging and biosensing. Microchim. Acta.

[134] Wu N., Liu X., Zeng M., Gao J., Lu X., Zeng Z., Zheng Y. (2019). Controllable synthesis of novel luminescent CuFeS_2_ quantum dots with magnetic properties and cation sensing features. J. Nanopart. Res..

[135] Zhao X., Lin C.W., Wang J., Oh D.H. (2014). Advances in Rapid Detection Methods for Foodborne Pathogens. J. Microbiol. Biotechnol..

[136] Al-Tayyar N.A., Youssef A.M., Al-hindi R. (2020). Antimicrobial food packaging based on sustainable Bio-based materials for reducing foodborne Pathogens: A review. Food Chem..

[137] Cho I.-H., Ku S. (2017). Current Technical Approaches for the Early Detection of Foodborne Pathogens: Challenges and Opportunities. Int. J. Mol. Sci..

[138] Xiong J., He S., Wang Z., Xu Y., Zhang L., Zhang H., Jiang H. (2022). Dual-readout fluorescence quenching immunochromatographic test strips for highly sensitive simultaneous detection of chloramphenicol and amantadine based on gold nanoparticle-triggered photoluminescent nanoswitch control. J. Hazard. Mater..

[139] Huang Z., Peng J., Han J., Zhang G., Huang Y., Duan M., Liu D., Xiong Y., Xia S., Lai W. (2019). A novel method based on fluorescent magnetic nanobeads for rapid detection of *Escherichia coli* O157:H7. Food Chem..

[140] Hu J., Jiang Y.-Z., Tang M., Wu L.-L., Xie H.-y., Zhang Z.-L., Pang D.-W. (2019). Colorimetric-Fluorescent-Magnetic Nanosphere-Based Multimodal Assay Platform for *Salmonella* Detection. Anal. Chem..

[141] Ghasemi R., Mirahmadi-zare S.Z., Nasr-Esfahani M.H., Allafchian A., Behmanesh M. (2019). Optical biosensing of *Streptococcus agalactiae* based on core/shell magnetic nanoparticle-quantum dot. Anal. Bioanal. Chem..

[142] Amaya-González S., De-los-Santos-Álvarez N., Miranda-Ordieres A.J., Lobo-Castañón M.J. (2013). Aptamer-Based Analysis: A Promising Alternative for Food Safety Control. Sensors.

[143] Du Y., Li B., Wang E. (2013). “Fitting” Makes “Sensing” Simple: Label-Free Detection Strategies Based on Nucleic Acid Aptamers. Acc. Chem. Res..

[144] Guo Z., Huang X., Li Z., Shi J., Zhai X., Hu X., Liang N., Zou X. (2020). Rapid and highly sensitive detection of *Salmonella typhimurium* in lettuce by using magnetic fluorescent nanoparticles. Anal. Methods.

[145] Li L., Li Q., Liao Z., Sun Y., Cheng Q., Song Y., Song E., Tan W. (2018). Magnetism-Resolved Separation and Fluorescence Quantification for Near-Simultaneous Detection of Multiple Pathogens. Anal. Chem..

[146] Ahmadian-Fard-Fini S., Salavati-Niasari M., Ghanbari D. (2018). Hydrothermal green synthesis of magnetic Fe_3_O_4_-carbon dots by lemon and grape fruit extracts and as a photoluminescence sensor for detecting of *E. coli* bacteria. Spectrochim. Acta Part B.

[147] Wang Z., Li X., Zhao Y., Yuan Y., Cai R., Yue T. (2018). Synthesis of multifunctional fluorescent magnetic nanoparticles for the detection of *Alicyclobacillus spp*. in apple juice. Food Res. Int..

[148] Cui W., Xu L., Shi Y., Dong N., Chen P. (2019). Rapid Detection of *Salmonella* via Off-on Composite Fluorescent Probe Based on Fluorescence Resonance Energy Transfer. Chin. J. Anal. Chem..

[149] Wall-Martinez H.A., Ramirez-Martinez A., Wesolek N., Brabet C., Durand N., Rodriguez-Jimenes G.C., Garcia-Alvarado M.A., Salgado-Cervantes M.A., Robles-Olvera V.J., Roudot A.C. (2019). Risk assessment of exposure to mycotoxins (aflatoxins and fumonisins) through corn tortilla intake in Veracruz City (Mexico). Food Addit. Contam. Part A.

[150] Tolosa J., Rodriguez-Carrasco Y., Ruiz M.J., Vila-Donat P. (2021). Multi-mycotoxin occurrence in feed, metabolism and carry-over to animal-derived food products: A review. Food Chem. Toxicol..

[151] Duracova M., Klimentova J., Fucikova A., Dresler J. (2018). Proteomic Methods of Detection and Quantification of Protein Toxins. Toxins.

[152] Wang C., Xiao R., Wang S., Yang X., Bai Z., Li X., Rong Z., Shen B., Wang S. (2019). Magnetic quantum dot based lateral flow assay biosensor for multiplex and sensitive detection of protein toxins in food samples. Biosens. Bioelectron..

[153] Shenashen M.A., Emran M.Y., El Sabagh A., Selim M.M., Elmarakbi A., El-Safty S.A. (2022). Progress in sensory devices of pesticides, pathogens, coronavirus, and chemical additives and hazards in food assessment: Food safety concerns. Prog. Mater. Sci..

[154] Li D., Qiao X., Lu J., Xu Z. (2018). Synthesis and Evaluation of a Magnetic Molecularly Imprinted Polymer Sorbent for Determination of Trace Trichlorfon Residue in Vegetables by Capillary Electrophoresis. Adv. Polym. Technol..

[155] Zhu W., Zhou Y., Liu S., Luo M., Du J., Fan J., Xiong H., Peng H. (2021). A novel magnetic fluorescent molecularly imprinted sensor for highly selective and sensitive detection of 4-nitrophenol in food samples through a dual-recognition mechanism. Food Chem..

[156] Hu Y., Liu J., Li J., Chen T., Wu M. (2018). Dual-functional imprinted magnetic nanoprobes for fluorescence detection of N-nitrosodiphenylamine. Anal. Methods.

[157] Shao Y., Wang Y., Yuan Y., Xie Y. (2021). A systematic review on antibiotics misuse in livestock and aquaculture and regulation implications in China. Sci. Total Environ..

[158] Lammie S.L., Hughes J.M. (2016). Antimicrobial Resistance, Food Safety, and One Health: The Need for Convergence. Annu. Rev. Food Sci. Technol..

[159] Li X., Li C., Chen L. (2015). Preparation of multifunctional magnetic–fluorescent nanocomposites for analysis of tetracycline hydrochloride. New J. Chem..

[160] Chen S., Su X., Yuan C., Jia C.Q., Qiao Y., Li Y., He L., Zou L., Ao X., Liu A. (2021). A magnetic phosphorescence molecularly imprinted polymers probe based on manganese-doped ZnS quantum dots for rapid detection of trace norfloxacin residual in food. Spectrochim. Acta Part A.

[161] Bunkoed O., Raksawong P., Chaowana R., Nurerk P. (2020). A nanocomposite probe of graphene quantum dots and magnetite nanoparticles embedded in a selective polymer for the enrichment and detection of ceftazidime. Talanta.

[162] Chansud N., Longnapa N., Bunkoed O. (2021). A nanohybrid magnetic sensing probe for levofloxacin determination integrates porous graphene, selective polymer and graphene quantum dots. J. Pharm. Biomed. Anal..

[163] Chaitong N., Chansud N., Orachorn N., Limbut W., Bunkoed O. (2021). A magnetic nanocomposite optosensing probe based on porous graphene, selective polymer and quantum dots for the detection of cefoperazone in milk. Microchem. J..

[164] Orachorn N., Bunkoed O. (2021). Nanohybrid magnetic composite optosensing probes for the enrichment and ultra-trace detection of mafenide and sulfisoxazole. Talanta.

[165] Xiao D., Jiang Y., Bi Y. (2018). Molecularly imprinted polymers for the detection of illegal drugs and additives: A review. Microchim. Acta.

[166] Huang Z., Xiong Z., Chen Y., Hu S., Lai W. (2019). Sensitive and Matrix-Tolerant Lateral Flow Immunoassay Based on Fluorescent Magnetic Nanobeads for the Detection of Clenbuterol in Swine Urine. J. Agric. Food Chem..

[167] Wu L., Lin Z.-Z., Zeng J., Zhong H.-P., Chen X.-M., Huang Z.-Y. (2018). Detection of malachite green in fish based on magnetic fluorescent probe of CdTe QDs/nano-Fe_3_O_4_@MIPs. Spectrochim. Acta Part B.

[168] Shi J., Zhang X., Zhang Q., Yang P. (2021). Ultrasensitive and Highly Selective Detection of Bisphenol a Using Core-Shell Magnetic Molecularly Imprinted Quantum Dots Electrochemiluminescent Probe. Bull. Environ. Contam. Toxicol..

